# Implementation and evaluation of the Y-Check comprehensive adolescent health check-up intervention in Zimbabwe: a pre−post mixed-methods study

**DOI:** 10.1038/s41591-025-04156-x

**Published:** 2026-02-02

**Authors:** Aoife M. Doyle, Farirai Nzvere, Salome Manyau, Victoria Simms, Ines Li Lin, Faith R. Kandiye, Chipo Ashley Nyamayaro, Michaela Takawira, Rudo M. S. Chingono, Mandikudza Tembo, Ronald Manhibi, Ethel Dauya, Chido Dziva Chikwari, Giulia Greco, Sarah Bernays, Valentina Baltag, Hannah Maisiri, Tonderai Kasu, Wenceslas Nyamayaro, Tsitsi Bandason, Constance R. S. Mackworth-Young, Prerna Banati, Helen A. Weiss, David A. Ross, Rashida A. Ferrand

**Affiliations:** 1 The Health Research Unit Zimbabwe, Biomedical Research and Training Institute, Harare, Zimbabwe; 2 International Statistics & Epidemiology Group, Department of Infectious Disease Epidemiology, London School of Hygiene & Tropical Medicine, London, UK; 3 Department of Global Health and Development, London School of Hygiene & Tropical Medicine, London, UK; 4 School of Public Health, University of Sydney, Sydney, New South Wales Australia; 5 Adolescent and Young Adult Health Unit, Department of Maternal Newborn, Child and Adolescent Health and Ageing, World Health Organization, Geneva, Switzerland; 6 Ministry of Primary and Secondary Education, Harare, Zimbabwe; 7 Ministry of Health and Child Care, Harare, Zimbabwe; 8 Institute for Life Course Health Research, Stellenbosch University, Stellenbosch, South Africa; 9 Department of Clinical Research, London School of Hygiene & Tropical Medicine, London, UK

**Keywords:** Population screening, Outcomes research

## Abstract

Routine adolescent health check-ups can support healthy development and well-being, but evidence on the feasibility, acceptability and effectiveness of contextually relevant comprehensive check-ups in low- and middle-income settings is limited. We conducted a hybrid implementation-effectiveness study incorporating a mixed-methods pre−post design of Y-Check, a comprehensive health check-up intervention in Zimbabwe, as part of a multicountry study developed and coordinated by the World Health Organization. Eligible participants were 10–19-year-old adolescents attending school or community venues. We used self-administered digital questionnaires, provider-led clinical tests and nurse reviews to screen for 25 conditions/behaviors. We provided health promotion, on-site care and referral to relevant providers. From October 2022 to September 2023, 2,097 adolescents were enrolled, of whom 1,843 (87.9%) were seen at 6 months. The primary outcome of appropriate care and/or referral(s) for all identified issues was achieved for 70.8% (95% confidence interval: 68.7–72.9%) of 1,865 participants with at least one issue. At follow-up, there were improvements in nutrition, health-related quality of life, self-esteem, behaviors and educational outcomes. The intervention was feasible and largely acceptable. Uptake of referral services varied by issue. Y-Check cost US$47 per participant. Through Y-Check, we identified untreated conditions and risk behaviors and successfully treated and linked adolescents to services. Here we provide evidence on the potential of the intervention to positively impact health and well-being.

## Abstract (web summary)

A WHO-supported pre−post study shows that implementation of the Y-Check comprehensive health check program is feasible and acceptable for adolescents in Zimbabwe, offering screening for 25 health conditions and behaviors, health promotion, on-site care and referral.

## Main

To accelerate progress toward achieving the Sustainable Development Goals (SDGs), the health and well-being of adolescents (10–19 years of age) needs to be improved^1–4^. The recently published second *Lancet* Commission on Adolescent Health and Well-being highlights that, although progress has been made globally in reducing the burden of disease for some conditions, insufficient progress has been made in reducing non-communicable diseases, with projected rises by 2030—for example, in the number of adolescents who are overweight and obese and in the burden of mental disorders and suicide^5^. In low- and middle-income countries (LMICs), improving access to preventive interventions is critical in the context of high morbidity and mortality from both communicable and non-communicable diseases^6^. African LMICs have the highest regional morbidity burden at 6,120 years of healthy life lost due to disability per 100,000 adolescents, with iron deficiency anemia and mental health disorders as the leading causes of morbidity^6^. Early and sustained engagement with health services can improve the health of adolescents, their adult health and the health of their offspring^6,7^. However, few adolescents have any contact with primary healthcare services, especially for health promotion and disease prevention, and services are not always appropriate for their needs^8^. The low priority and limited investment in adolescent health in African LMICs has led to great health condition disparity in the quality and availability of services. Going forward, it is imperative that adolescent health services be developed that address the comprehensive needs of adolescents with financing that matches the disease burden^5,9^.

Schools offer an important opportunity to reach adolescents with health services^10^, and routine health check-ups (‘well-care visits’) offer a potential solution^8,11–13^. Well-care visits are scheduled routine, regular check-ups by healthcare providers to ensure the healthy growth, development and well-being of adolescents^13^. The WHO and partners are developing guidelines on preventive and promotive contacts with the health system for adolescents; however, evidence on the feasibility, acceptability and effectiveness of routine health check-ups in LMICs is scarce. Most routine school health screening programs in LMICs focus on malnutrition, vision, hearing and oral health and exclude other important adolescent concerns, such as psychosocial issues, mental health, substance use and sexually transmitted infections (STIs)^10,14^.

**Fig. 1:**
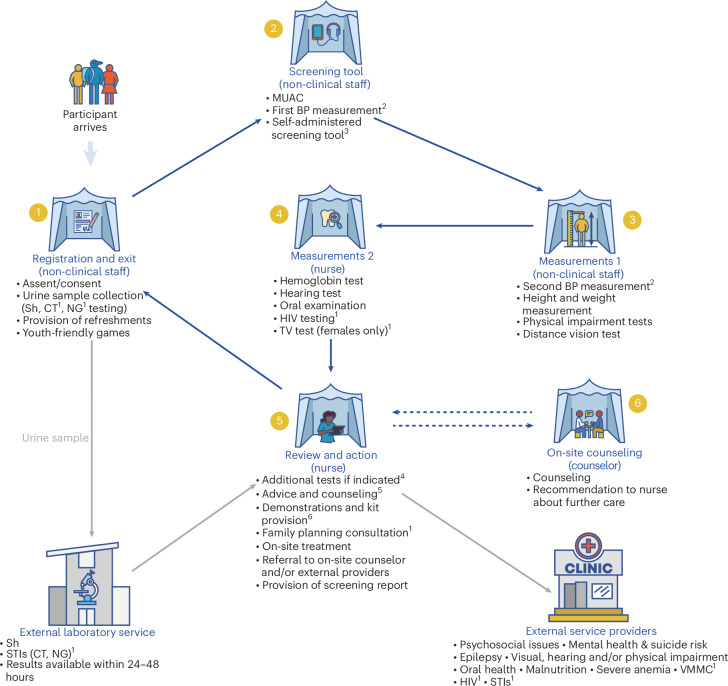
Y-Check intervention. Flowchart showing the participant flow and services provided during the check-up visit. ^a^Community only (16–19 years). ^b^15–19 years. ^c^HEEADSSS, oral hygiene, sleep and epilepsy. ^d^Third BP measurement, ear exam +/– wax removal. ^e^Oral hygiene, sleep, physical activity, mental health and nutrition. ^f^Oral care, menstrual hygiene and health (females only), and condoms (16–19 years, community only). Sh, *Schistosoma haematobium*; CT, *Chlamydia trachomatis*; NG, *Neisseria gonorrhoeae*; MUAC, mid-upper arm circumference; BP, blood pressure; HEEADSSS, home environment, education/employment, eating, peer-related activities, drugs, sexuality, suicide/depression and safety; TV, *Trichomonas vaginalis*; VMMC, voluntary medical male circumcision; HIV, human immunodeficiency virus; STI, sexually transmitted infection.

To address this evidence gap, the WHO is coordinating the Y-Check research program, with studies of check-ups in Zimbabwe, Tanzania and Ghana^15–18^. Y-Check provides screening, treatment and/or referral of adolescents for 25 high-burden conditions and risk behaviors through check-up visits at WHO-recommended timepoints: early adolescence (10–14 years) and mid/late adolescence (15–19 years)^15^. The Y-Check approach is novel and innovative as, compared to other adolescent health check-up programs, it provides a more comprehensive check-up with target conditions and services informed by local epidemiology, evidence on effective interventions and stakeholder priorities (P.B., A.M.D., S.K., B.W., M.V., E.A., F.G., M.K.N., F.P.N., V.B., H.A.W., R.A.F. and D.A.R. for the Y-Check research program team. Identifying the content of an adolescent health and well-being screening program: evidence from Y-Check. International Association for Adolescent Health World Congress 2025)^16–18^. Y-Check focuses on conditions with accurate, acceptable, affordable screening tests and locally accessible, affordable interventions (for example, mental health, HIV, vision, hearing and anemia). General health information, risk reduction and psychosocial counseling are also provided (Fig. 1).

The Y-Check intervention was developed and evaluated first in Zimbabwe in collaboration with WHO. Contextual adaptation, implementation and evaluation are ongoing in Tanzania and Ghana. Zimbabwe is a lower-middle-income country in southern Africa with a population of 16.5 million (ref. ^19^) and a median age of 18 years. It is ranked 137 out of 167 countries based on its performance across the SDGs and is on track to achieving only two (new HIV infections and routine immunization of infants) of the 14 SDG3 (Good Health and Well-Being) indicators. In 2021, Zimbabwe had a Universal Health Coverage index of 55, which is above the average for African countries of 44 but well below the target of 100 by 2030 (ref. ^20^). Adolescents can access primary healthcare services at nurse-led primary healthcare clinics; however, clinics face challenges with workforce retention, drug and equipment supplies and maintenance of electricity, water and sanitation services. External donor funding has led to siloed vertical services resulting, for example, in free HIV and sexual and reproductive health services for adolescents but more resource-constrained non-communicable disease services subject to a user fee (approximately US$5 per visit)^18,21^. Zimbabwe is making moderate progress toward achievement of the SDG4 goal of Quality Education, but major challenges remain^22^, and investment in the education systems in Zimbabwe is lower than the recommended 4–6% of GDP^23^. In 2024, completion rates were estimated to be 87% for primary school (Grades 1–7), 74% for the first half of secondary school (Forms 1–4, 13–16 years) and 8% for the second half of secondary school (Forms 5–6, 17–18 years)^24^.

We report here on the hybrid effectiveness-implementation study through which we assessed Y-Check’s feasibility and effects on adolescent health and well-being in Chitungwiza, Zimbabwe’s third-largest city.

## Results

### Sociodemographic characteristics

**Extended Data Fig. 1:**
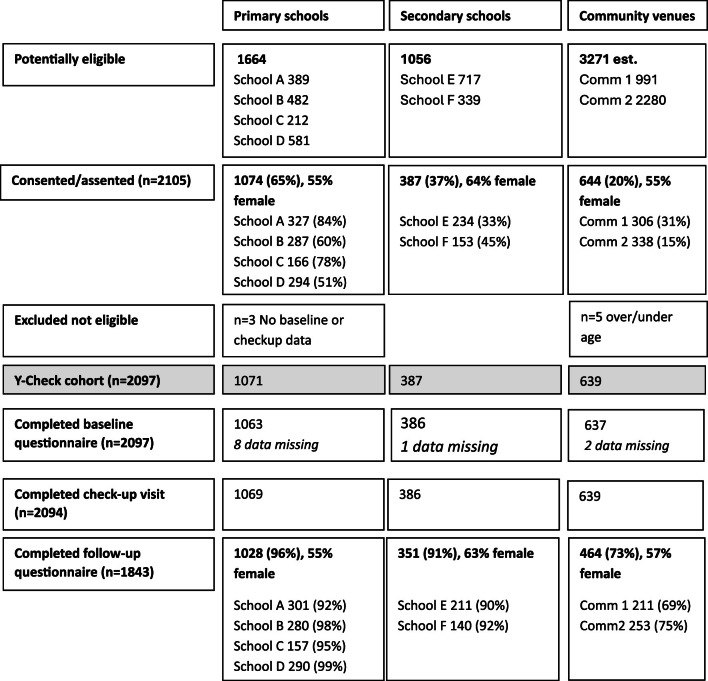
Study profile. Flowchart showing recruitment and follow-up of study participants.

Students in Grade 5 from four primary schools (*n* = 1,664) and in Form 3 from two secondary schools (*n* = 1,056) were invited to receive the intervention. Of these, 1,071 (64.4%) primary students and 387 (36.6%) secondary students were eligible. The main reason for ineligibility was lack of assent and/or consent. In addition, 639 participants from an estimated population of 3,271 potentially eligible community-based adolescents were enrolled at two community venues. Community venues were multipurpose use buildings or community centers. Between 14 October 2022 and 23 March 2023, 2,097 participants were enrolled and completed the baseline evaluation questionnaire, of whom 2,094 received the check-up visit (Extended Data Fig. 1).

Overall, 56.5% of participants were female. The mean age was 11.1 years (s.d. 0.7, range 10–14) in primary schools, 16.2 years (s.d. 0.8, range 15–19) in secondary schools and 16.6 years (s.d. 0.6, range 16–19) at community venues. Among participants recruited at community venues, 155 (24.3%) were not enrolled in school. 84.7% were living with at least one parent and 81.4% of guardians were employed (Table 1).

**Table 1:** Participant characteristics by sex and check-up location

		Male (*n* = 912)	Female (*n* = 1,185)	Primary schools (*n* = 1,071)	Secondary schools (*n* = 387)	Community venues (*n* = 639)
Age in years	10–11	368 (40.4%)	476 (40.2%)	844 (78.8%)		
	12–13	115 (12.6%)	105 (8.9%)	220 (20.5%)		
	14–15	14 (1.5%)	56 (4.7%)	7 (0.7%)	63 (16.3%)	
	16–17	378 (41.4%)	503 (42.4%)		294 (76.0%)	587 (91.9%)
	18–19	37 (4.1%)	45 (3.8%)		30 (7.7%)	52 (8.1%)
Sex	Male			485 (45.3%)	140 (36.2%)	287 (44.9%)
	Female			586 (54.7%)	247 (63.8%)	352 (55.1%)
Location	School A	128 (14.0%)	198 (16.7%)	326 (30.4%)		
	School B	134 (14.7%)	151 (12.7%)	285 (26.6%)		
	School C	83 (9.1%)	83 (7.0%)	166 (15.5%)		
	School D	140 (15.4%)	154 (13.0%)	294 (27.5%)		
	School E	84 (9.2%)	150 (12.7%)		234 (60.5%)	
	School F	56 (6.1%)	97 (8.2%)		153 (39.5%)	
	Community 1	158 (17.3%)	147 (12.4%)			305 (47.7%)
	Community 2	129 (14.1%)	205 (17.3%)			334 (52.3%)
Who the participant lives with (multiple selection)	Parents +/− other relatives	794 (87.1%)	981 (82.8%)	970 (90.6%)	315 (81.4%)	490 (76.7%)
	Other relatives (not parents)	111 (12.2)	196 (16.5%)	100 (9.3%)	70 (18.1%)	137 (21.4%)
	Partner/spouse (not relatives)	0 (0%)	6 (0.5%)	1 (0.1%)	0 (0%)	5 (0.8%)
	Lives alone	7 (0.8%)	2 (0.2%)	0 (0%)	2 (0.5%)	7 (1.1%)
Guardian working status	Working for someone (self-employed and employed)	757 (83.4%)	942 (80.0%)	878 (82.6%)	325 (84.2%)	496 (77.9%)
	Unemployed (seeking and not seeking work)	90 (9.9%)	177 (15.0%)	119 (11.2%)	42 (10.9%)	106 (16.6%)
	Retired	21 (2.3%)	27 (2.3%)	20 (1.9%)	13 (3.4%)	15 (2.4%)
	Unknown (do not know/do not want to answer)	40 (4.4%)	32 (2.7%)	46 (4.3%)	6 (1.5%)	20 (3.1%)
	Missing	4	7	8	1	2
Asset index	1st (lowest quintile)	135 (17.3%)	225 (22.2%)	162 (18.7%)	65 (18.5%)	133 (23.1%)
	2nd	147 (18.8%)	216 (21.3%)	165 (19.0%)	78 (22.2%)	120 (20.9%)
	3rd	151 (19.4%)	220 (21.7%)	169 (19.5%)	84 (23.9%)	118 (20.5%)
	4th	173 (22.2%)	187 (18.4%)	181 (20.9%)	69 (19.7%)	110 (19.1%)
	5th (highest quintile)	173 (22.2%)	166 (16.4%)	190 (21.9%)	55 (15.7%)	94 (16.4%)
	Excluded	129	164	196	35	62
	Missing	4	7	8	1	2
Religion	Muslim	28 (3.1%)	42 (3.6%)	60 (5.6%)	3 (0.8%)	7 (1.1%)
	Christian	782 (86.1%)	1036 (87.9%)	888 (83.5%)	363 (94.0%)	567 (89.0%)
	African traditional	26 (2.9%)	27 (2.3%)	30 (2.8%)	7 (1.8%)	16 (2.5%)
	None	28 (3.1%)	34 (2.9%)	32 (3.0%)	8 (2.1%)	22 (3.5%)
	Do not know	29 (3.2%)	24 (2.0%)	39 (3.7%)	2 (0.5%)	12 (1.9%)
	Other	15 (1.7%)	15 (1.3%)	14 (1.3%)	3 (0.8%)	13 (2.0%)
	Missing	4	7	8	1	2

The assets: refrigerator, bicycle, animal-drawn cart, motorcycle, boat, cart, television, radio, microwave, cell phone and computer. We excluded everyone who said they owned either zero assets (*n* = 108) or all 11 assets (*n* = 185), because the data presented ceiling and floor effects distribution, which is unlikely given the socioeconomic context of the study locations. We performed a factor analysis for data reduction and generation of the asset index. Each asset gets a ‘weight’ (factor loading), and patterns of asset ownership predict the asset index score. The scores are grouped into quintiles.

Between 1 June and 13 September 2023, 1,843 (87.9%) adolescents participated in the follow-up evaluation survey (Extended Data Fig. 1). The median time to follow-up was 5.5 months (interquartile range 5.0–7.1). Follow-up was higher among adolescents in schools (94.6%) than among those in the community (72.6%) and was similar across sociodemographic characteristics, except at community venues where follow-up was lower among males and those out of school (Supplementary Table 1). The main reasons for loss to follow-up were absence on the visit day or transfer to another school (school participants) or relocation/not contactable on household visits (community participants).

### Screening results

During the check-up visits, screening data entered into the Y-Check application led to the real-time condition-specific and behavior-specific warning flags that nurses reviewed and actioned. During the review process, some warning flags were deemed incorrect—for example, if the adolescent had misunderstood the question. Overall, 1,865 (89.1%) participants screened positive for at least one issue, and 669 referrals of 565 participants (30.3% of those screening positive) were made to external service providers. The prevalence of untreated conditions and risk behaviors was similar for males and females but higher among older (15–19-year-old) than younger (10–14-year-old) adolescents (94.8% versus 79.1%, *P* < 0.001) (Table 2). This was only partly due to more issues being screened for in the older adolescents.

**Table 2:** Yield of untreated conditions and prevalence of risk behaviors at check-up by sex and age group

Screening method	Condition/behavior^a^	Overall (*n* = 2,094)	Ages 10–14 (primary schools)		Ages 15–19 (secondary schools and community venues)	
			Males (*n* = 485)	Females (*n* = 584)	Males (*n* = 426)	Females (*n* = 599)
Self-report	Psychosocial issues^b^	848/2,094 (40.5%)	185/485 (38.1%)	209/584 (35.8%)	175/426 (41.1%)	279/599 (46.6%)
	Limited physical activity	829/2,094 (39.6%)	137/485 (28.2%)	199/584 (34.1%)	133/426 (31.2%)	360/599 (60.1%)
	Poor oral hygiene	385/2,094 (18.4%)	134/485 (27.6%)	130/584 (22.3%)	68/426 (16.0%)	53/599 (8.8%)
	Alcohol use	369/2,094 (17.6%)	42/485 (8.7%)	19/584 (3.3%)	170/426 (39.9%)	138/599 (23.0%)
	Poor sleep	286/2,094 (13.7%)	36/485 (7.4%)	35/584 (6.0%)	72/426 (16.9%)	143/599 (23.9%)
	Suicide risk	268/2,094 (12.8%)	29/485 (6.0%)	44/584 (7.5%)	56/426 (13.1%)	139/599 (23.2%)
	Common mental health disorders	243/2,094 (11.6%)	34/485 (7.0%)	36/584 (6.2%)	58/426 (13.6%)	115/599 (19.2%)
	Drug use	82/2,094 (3.9%)	8/485 (1.6%)	7/584 (1.2%)	48/426 (11.3%)	19/599 (3.2%)
	Smoking	57/2,094 (2.7%)	3/485 (0.6%)	0/584 (0.0%)	43/426 (10.1%)	11/599 (1.8%)
	Epilepsy	9/2,094 (0.4%)	2/485 (0.4%)	5/584 (0.9%)	0/426 (0.0%)	2/599 (0.3%)
Self-report (community venues only)	VMMC need	145/274 (52.9%)	N/A	N/A	145/274 (52.9%)	N/A
	Family planning need	82/155 (52.9%)	N/A	N/A	36/83 (43.4%)	46/72 (63.9%)
	Sexual risk	87/639 (13.6%)	N/A	N/A	51/287 (17.8%)	36/352 (10.2%)
	STI symptoms	19/639 (3.0%)	N/A	N/A	4/287 (1.4%)	15/352 (4.3%)
	HIV+ and not engaged in care	10/639 (1.6%)	N/A	N/A	6/287 (2.1%)	4/352 (1.1%)
Physical examination/ measurement	Elevated blood pressure^d^	319/966 (33.0%)	N/A	N/A	140/398 (35.2%)	179/568 (31.5%)
	Oral health condition^e^	234/2,070 (11.3%)	44/479 (9.2%)	56/575 (9.7%)	55/421 (13.1%)	79/595 (13.3%)
	Visual impairment	145/2,091 (6.9%)	26/485 (5.4%)	35/584 (6.0%)	32/426 (7.5%)	52/596 (8.7%)
	BMI, overweight	211/2,094 (10.1%)	42/485 (8.7%)	61/584 (10.4%)	8/426 (1.9%)	100/599 (16.7%)
	BMI, underweight	98/2,094 (4.7%)	31/485 (6.4%)	22/584 (3.8%)	38/426 (8.9%)	7/599 (1.2%)
	BMI, obesity	53/2,094 (2.5%)	11/485 (2.3%)	24/584 (4.1%)	3/426 (0.7%)	15/599 (2.5%)
	Physical impairment^c^	25/2,094 (1.2%)	2/485 (0.4%)	7/584 (1.2%)	11/426 (2.6%)	5/599 (0.8%)
	Hearing impairment^f^	10/2,059 (0.5%)	2/480 (0.4%)	3/576 (0.5%)	4/410 (1.0%)	1/593 (0.2%)
Laboratory test	Anemia^g^	244/2,073 (11.8%)	29/480 (6.0%)	56/577 (9.7%)	38/421 (9.0%)	121/595 (20.3%)
	Schistosoma haematobium^h^	49/2,077 (2.4%)	12/479 (2.5%)	7/582 (1.2%)	22/424 (5.2%)	8/592 (1.4%)
	STI, CT/NG test^i,l^	36/630 (5.7%)	N/A	N/A	6/282 (2.1%)	30/348 (8.6%)
	STI, TV test^j,l^	21/344 (6.1%)	N/A	N/A	N/A	21/344 (6.1%)
	HIV test^k^^,l^	4/606 (0.7%)	N/A	N/A	2/276 (0.7%)	2/330 (0.6%)
	Any conditions/risk behaviors	1,818/2,094 (86.8%)	387/485 (79.8%)	459/584 (78.6%)	404/426 (94.8%)	568/599 (94.8%)

^a^Definitions of conditions and behaviors are provided in Supplementary Table 4. ^b^Psychosocial issues are a combination of Home, School/Work, Body, Meals and Friends flags. There were 142 participants who reported not in school or working, excluded in the denominator for the School/Work flag but included in the Psychosocial issues denominator. ^c^Physical impairment is a combination of Grip and Jump test flags. All participants were tested for at least one physical impairment test. There are 22 missing jump test measurements and 10 missing grip measurements. Physical impairment denominator includes all participants who have data on at least one of the flags that make the combined measure. ^d^Blood pressure, 59 missing data (28 males, 31 females); ^e^oral health, 24 missing data (aged 10–14: 6 males, 9 females; aged 15–19: 5 males, 4 females); ^f^hearing, 35 missing data (aged 10–14: 5 males, 8 females; aged 15–19: 16 males, 6 females); ^g^anemia, 21 missing data (aged 10–14: 5 males, 7 females; aged 15–19: 5 males, 4 females); ^h^schistosomiasis, 17 missing data (aged 10–14: 6 males, 2 females; aged 15–19: 2 males, 7 females); ^i^STI, CT/NG, 9 missing data (aged 15–19: 5 males, 4 females); ^j^STI, *Trichomonas vaginalis* (TV), (aged 15–19: 8 missing data females); ^k^HIV test, 12 refused test and 21 reported positive on antiretroviral therapy, thus not tested for HIV. The remaining 161 with missing data (158 males, 3 females) were assumed to have tested negative and were included in the denominator as the nurse field notes did not identify them as having tested positive or as having refused testing. ^l^Only tested among participants attending the community venues.

In younger adolescents (10–14 years), the only condition with a prevalence of 10% or more was psychosocial issues (37.2%) (Table 2). Among older adolescents (15–19 years), the most prevalent conditions were psychosocial issues (40.5%), elevated blood pressure (33.0%), suicide risk (19.0%), common mental disorders (CMDs) (16.9%), anemia (15.7%) and oral health conditions (13.2%). Nutritional assessments showed that 7.6% of males and 2.5% of females were underweight, and 1.5% of males and 3.3% of females were obese (Table 2). Only 9.1% and 15.6% of females reported having received one or two doses of the HPV vaccine, respectively.

The most prevalent risk behaviors were limited physical activity (39.6%) and poor oral hygiene (18.4%). Among older adolescents, alcohol use (30.0%) and poor sleep (21.0%) were also prevalent. Among the older adolescents (community venue only), 13.6% reported engaging in high-risk sexual behavior, and 52.9% of males were uncircumcised (Table 2).

### Short-term care outcomes

**Fig. 2:**
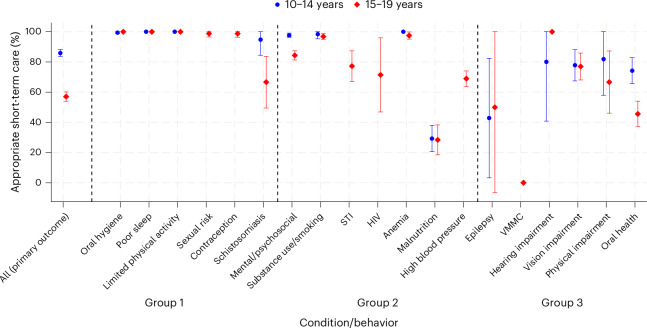
Proportion (95% confidence interval) of participants screening positive for the issue who received appropriate on-the-spot care and/or completed appropriate referral before the date of the follow-up visit by issue. Data are presented for total population of *n* = 2,094 adolescents (*n* = 1,069 10–14 years, *n* = 1,025 15–19 years). Vertical gray lines separate issues (conditions/behaviors) by care management groups: Group 1, on-the-spot care only; Group 2, on-the-spot care with referral if necessary; Group 3, referral with or without on-the-spot care. Error bars represent 95% confidence intervals. Definitions of conditions and behaviors are provided in Supplementary Table 4.

For the primary outcome, 1,321 of 1,865 (70.8%; 95% confidence interval: 68.7−72.9%) participants who screened positive for at least one issue (condition/behavior) had received appropriate short-term care by the time they were followed-up. The proportion receiving appropriate short-term care varied substantially by age group and sex (Extended Data Table 1) and by issue (Fig. 2). For issues that were managed primarily or exclusively on the spot, the proportions receiving appropriate short-term care were all greater than 90%. These issues included psychosocial issues, CMDs, substance use/smoking, poor oral hygiene, poor sleep, limited exercise, sexual risk, family planning and anemia. For issues that usually required referral to government or non-governmental organization service providers, the proportion receiving appropriate short-term care varied from 0.0% for voluntary medical male circumcision (VMMC) to 90% for hearing impairment (Fig. 2 and Supplementary Table 2).

Sensitivity analysis showed that the overall proportion receiving appropriate on-the-spot care was 85.3% (95% confidence interval: 83.6−86.8%). Restricting the primary outcome definition to documented referral attendance within 4 months decreased this proportion (61.6%; 95% confidence interval: 59.3−63.7%), and broadening it to include self-reported referral attendance increased it (73.2%; 95% confidence interval: 71.1−75.2%) (Extended Data Table 1). After implementation, we identified individuals who had been erroneously flagged during the check-up. Excluding these, 1,300 of 1,833 (70.9%; 95% confidence interval: 68.8−73.0%) individuals received appropriate short-term care. When restricting the analysis to issues applicable to all ages and sex, by excluding sexual and reproductive conditions and blood pressure, 1,446 of 1,806 (80.1%; 95% confidence interval: 78.2−81.8%) individuals received appropriate short-term care, and differences according to sex were no longer present. Excluding previously diagnosed issues that did not require further management did not change the primary outcome because all nine participants affected also had another previously undiagnosed issue.

### Clinical outcomes

**Fig. 3:**
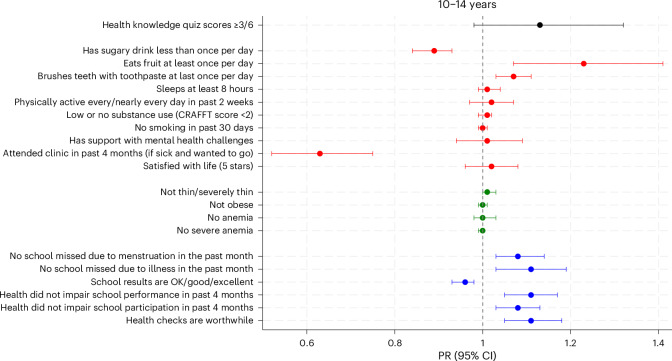
Relative difference in individual outcomes among 10–14-year-olds (*n* = 1,028) comparing before and after the intervention (prevalence ratio and 95% confidence interval). Prevalence ratio (PR) was derived from generalized linear models fitting population-averaged panel data, adjusted for within-location correlation. A PR greater than 1.0 indicates an improvement at follow-up. Error bars represent 95% confidence intervals (CIs). Definitions of evaluation outcomes are provided in Supplementary Table 5. CRAFFT, Car, Relax, Alone, Forget, Friends/Family, Trouble questionnaire for substance use risk in adolescents. ‘Health checks are worthwhile’ was defined as rating 5 on a scale of 1–5 in response to the question ‘How worthwhile do you think getting your health checked is?’. See Supplementary Table 3a,c for further details on each secondary outcome prevalence at baseline and at follow-up, difference in proportions and PR (95% CI).

**Fig. 4:**
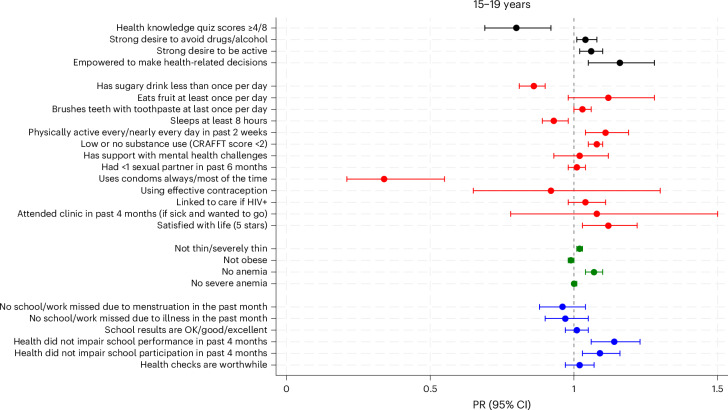
Relative difference in individual outcomes among 15–19-year-olds (*n* = 815) comparing before and after the intervention (prevalence ratio and 95% confidence interval). PR was derived from mixed-effects generalized linear model fitting population-averaged panel data, adjusting for the random effects of location. A PR greater than 1.0 indicates an improvement at follow-up. Error bars represent 95% CIs. Definitions of evaluation outcomes are provided in Supplementary Table 5. CRAFFT, Car, Relax, Alone, Forget, Friends/Family, Trouble questionnaire for substance use risk in adolescents. Condom use and effective contraception were asked only in sexually active adolescents in the community setting; effective contraception outcome is shown for females only. ‘Health checks are worthwhile’ was defined as rating 5 on a scale of 1–5 in response to the question ‘How worthwhile do you think getting your health checked is?’. See Supplementary Table 3b,d for further details on each secondary outcome prevalence at baseline and at follow-up, difference in proportions and PR (95% CI).

Malnutrition and anemia were assessed in all participants at baseline and follow-up. Comparing baseline and follow-up prevalence showed that the prevalence of thinness decreased between check-up and follow-up in both age groups. In 15−19-year-olds, anemia decreased, whereas obesity increased (Figs. 3 and 4 and Supplementary Table 3). In those who screened positive for a clinical condition at check-up and were reassessed at follow-up, improvement in symptoms was observed for STI symptoms (93.3% improved, 14/15), *Chlamydia trachomatis* and/or *Neisseria gonorrhoeae* (CT/NG) (66.7%, 20/30), *Trichomonas vaginalis* (81.8%, 9/11), CMD symptoms (91.0%, 181/199), suicide ideation (82.7%, 177/214), visual impairment (56.1%, 37/66) and hearing impairment (100%, 10/10) (Extended Data Table 2).

### Health knowledge and behaviors

There was evidence of a decrease in health-related knowledge among older females (29.5% to 22.5%, *P* = 0.005) but no difference for the other age/sex groups (Figs. 3 and 4 and Supplementary Table 3). There was evidence of increases in some reported healthy behaviors (toothbrushing (for example, younger males 72.8% to 77.4%, *P* = 0.03), fruit consumption (for example, younger females 24.6% to 29.9%, *P* = 0.03) and physical activity (for example, older females 40.7% to 48.4%, *P* = 0.002)) and decreases in risk behaviors (low or no substance use (for example, older males 81.7% to 91.1%, *P* < 0.001)). However, there was a decrease in those reporting low levels of sugary drink consumption (for example, older males 84.5% to 72.5%, *P* < 0.001) and a decrease in healthy sleep habits among older males (81.7% to 71.6%, *P* = 0.003). There was evidence of a reduction in the proportion of younger adolescents who reported that they had attended a clinic within the past 4 months when they were sick and wanted to go (for example, younger males 65.1% to 40.7%, *P* < 0.001). There was no change in the proportion who reported that they had support for mental health challenges. Among the older adolescents who reported that they were sexually active in the past 6 months, use of condoms most or all of the time decreased in the follow-up period (for example, older males 66.2% to 17.0%, *P* < 0.001), with no change in the reported number of sexual partnerships, effective contraceptive use or in linkage to HIV care among those who reported that they were HIV+ (Figs. 3 and 4 and Supplementary Table 3).

### Health-related intentions and self-efficacy, quality of life, self-esteem and life satisfaction

Health-related intentions (desire to avoid drugs/alcohol or to be active) and self-efficacy (feeling empowered to make health-related decisions), measured in older adolescents only, increased over time. There was an increase in the proportion reporting that health checks were worthwhile among younger adolescents (Figs. 3 and 4 and Supplementary Table 3). Overall, there was strong evidence of an improvement in mean self-esteem score (29.2 to 30.2, *P* < 0.001) and a decrease in the proportion with a low self-esteem score (<25) (14.8% to 8.5%; *P* < 0.001) (Extended Data Table 3). The overall mean health-related quality of life score also improved slightly from 0.83 to 0.86 (*P* < 0.001) (Extended Data Table 3). Life satisfaction improved among older females (40.9% to 48.2%, *P* = 0.01) but not older males.

### Educational outcomes

Positive changes were observed in some self-reported educational outcomes, with days of school missed due to menstruation or illness decreasing in younger adolescents and impairment of performance and participation due to poor health decreasing in all four age/sex groups. However, among younger adolescents, there was a decrease in the proportion who reported that their school results were ‘OK’, ‘good’ or ‘excellent’ (Figs. 3 and 4 and Supplementary Table 3).

### Process evaluation findings

#### Feasibility

It was feasible to screen for multiple issues at the same check-up visit, although intervention staff reported high workload. Y-Check staff reported that the self-completion questionnaire increased sensitivity through an increased sense of confidentiality and may have reduced reporting bias, and the nurse review increased specificity by identifying false warning flags. However, some adolescents struggled to understand and/or maintain concentration when self-completing the questionnaire, and 16% of younger and 4% of older adolescents needed assistance from a staff member. Where the number of false flags was high, the visit was less efficient due to time discussing non-issues.

#### Fidelity

Intervention fidelity was high: 91.8% of participants received all the planned screening. Programming the digital application was complex, and residual errors in algorithms hindered implementation and fidelity.

Establishing appropriate, high-quality and available referral pathways was the primary challenge, and there were delays in establishing some referral pathways: ‘The parents were getting restless and worried, yet they could not get any assistance because the referral system was not yet in place’ (interview, intervention staff). In many cases, appropriate government services were either not available or not acceptable to adolescents and their families. Some improvements were made during implementation—for example, adding a Y-Check referral focal person to coordinate referrals.

#### Adoption

Observations of the engagement and consenting processes revealed that parental and teacher support was key to uptake and was notably higher in primary schools. Some parents of secondary school learners were resistant to the health intervention happening during school hours: ‘My parents said no, school is for education, and I should stick to education’ (workshop, male adolescent, 16 years old). Although considerable Y-Check resources were invested in community and school engagement, stakeholders suggested that it should be broadened further to include all school staff, and even more intensive community engagement would be required to maximize Y-Check impact.

Other barriers to intervention uptake included cultural and religious misconceptions around blood and urine collection and satanism and fears that Y-Check may diagnose conditions but fail to provide treatment and burden parents with healthcare costs. The inclusion of sexual and reproductive health services for participants at the community venues increased uptake; in particular, condoms were popular among males.

Referral uptake was low for oral health, mental health and malnutrition, in particular obesity. Some issues were considered by adolescents and their families not to be important, and they did not always believe that the appropriate referral service would be available.

Adoption of some healthier behaviors, such as having a balanced and adequate diet, was restricted by limited family finances, which had to be split among housing costs, school fees and food for the family.

#### Acceptability

Most (84%) participants reported being ‘very satisfied’ at the exit interview (Extended Data Table 4). Adolescents particularly appreciated the youth-friendly service delivery and being able to check if they were healthy: ‘There is no other program I know that can make you feel comfortable talking about issues. She listened and I was able to explain everything’ (interview, male adolescent, 17 years old).

Adolescents who suspected that they had a health problem, or had a known but unmanaged issue, appreciated that Y-Check could provide a diagnosis and initial care: ‘I actually thought I had a problem with my ears, and I was happy to be checked so that I know, and it can be helped’ (workshop, adolescent male, 16 years old).

Adolescents who had attended the check-ups reported that they liked the fact that services were free, as user fees are payable at government health facilities. Adolescents appreciated having their health checked and receiving comprehensive quality services in a youth-friendly setting. The waiting time was thought to be long but mitigated by the availability of games while waiting. Teachers generally appreciated Y-Check as it would lessen the burden of adolescent ill-health on their parents, who could not afford it. Although many parents, particularly those of primary school children, appreciated the support from Y-Check, some parents feared that the check-ups would diagnose new conditions in their children, adding the burden of long-term healthcare costs to other competing priorities of school fees, food and health needs of other family members.

#### Appropriateness

For younger adolescents, holding check-ups in school was seen as appropriate, but, for older adolescents, community settings were seen as more appropriate, largely due to confidentiality: ‘I said to myself, what if they find that my child has a problem and the whole school gets to know about it, so I refused to get my child checked there’ (workshop, parent of secondary school adolescent).

All stakeholders raised concerns about the sustainability of continued care and health benefits given adolescents’ limited access to ongoing preventive and curative health services. For example, the provision of a one-off supply of a toothbrush and toothpaste, and of reusable menstrual pads, with no future supply was felt to be challenging given the economic context: ‘We know they don’t have the money for toothpaste. After Y-Check, how can they continue brushing their teeth?’ (interview, intervention staff). Intervention staff expressed concerns in addressing malnutrition among adolescents through referral to a nutritionist, without addressing the family’s economic circumstances: ‘I teach them about a balanced diet, yet most of them have no food to cook. I send them away with Plumpy’Nut for one child yet the whole family is hungry’ (interview, service provider).

#### Cost

The total cost of delivering Y-Check was US$423,301, of which US$202,169 was for setup (intervention design and piloting) and US$221,133 was for implementation. Excluding setup costs, the average cost to screen and treat an adolescent was US$38. The average cost to screen, treat and/or refer was US$47 per adolescent.

## Discussion

Comprehensive adolescent health check-ups were feasible and potentially acceptable in this resource-limited, urban African context. Acceptability was high among adolescents who participated in primary schools and community settings and among those who attended the check-ups in all three settings. However, uptake of the intervention was low in secondary schools largely due to a low proportion of parents providing their written opt-in consent. Adolescents, guardians and teachers found the intervention to be acceptable and highly valued its youth-friendliness and the fact that the services were free. We identified a substantial untreated burden of disease among the adolescents using relatively simple and cheap methods. Overall, 89% of adolescents screened had at least one untreated condition or risk behavior. Appropriate on-the-spot care and/or completed referral was achieved for 70.8% of those with an untreated condition or risk behavior. At follow-up, we observed decreased thinness, decreased prevalence of anemia among 15–19-year-old females and improvements in many clinical conditions, health-related quality of life, some educational outcomes, self-efficacy, self-esteem and four of eight self-reported behaviors.

The intervention was innovative as it strove to address the comprehensive health needs of adolescents and provided treatment and health promotion/prevention activities for 25 issues (conditions/behaviors) in one visit. On site, we were able to screen adolescents and provide immediate care for almost all issues, with only 30.3% of those screening positive requiring referral. A strength of the study was that it evaluated an intervention whose content had been rigorously developed, including co-development with adolescents. Co-design activities included a crowdsourcing contest^25^, cognitive interviews and linguistic validation workshops to refine the wording of the screening tools^26^. Participants reported that the resultant intervention was youth-friendly, context relevant, acceptable to other stakeholders and feasible to implement. The intervention successfully reached adolescents who are particularly hard to reach: males and out-of-school youth^27,28^.

The use of digital technologies to facilitate cost-effective health systems and services is a core objective of the WHO Global Strategy on Digital Health^29^. A novel aspect of intervention delivery was the creation and use of a bespoke digital application, which enabled on-the-spot generation of warning flags for health conditions and risk behaviors, which the nurse could review and action. The youth-friendly digital application increased visit efficiency. In this setting, we would recommend that assistance is available for the relatively small proportion of participants with low levels of literacy and/or who are not familiar with digital tools who will struggle with the self-completion questionnaire. This is especially important for those in primary school. Given the complexity of programming the application for this multicondition check-up visit, for future similar interventions, we recommend an extended period of development and testing of digital data collection and screening tools.

Y-Check represents a novel implementation strategy to improve adolescent access to evidence-based interventions. In the present study, a contemporaneous control group in which conditions and risk behaviors were screened for at baseline would not have been ethical because, in ‘control’ populations, the primary implementation outcome could only be measured through the delivery of the equivalent of Y-Check that is screening and then treatment/referral for identified issues. Notably, the prospective pre−post intervention design was strengthened by there being a counterfactual, because baseline screening mainly identified undiagnosed or untreated chronic conditions, which indicated what would have occurred in the absence of the intervention. Qualitative process evaluation data further strengthened inference. For example, the observed positive impact on anemia and small reduction in thinness but lack of impact on obesity is plausible given reported acceptability and uptake of supplementation services for underweight and anemic adolescents but limited services available for those who were obese, in addition to social norms that value larger body size^30,31^.

The cost per adolescent of US$47 is relatively high when compared to Zimbabwe’s per capita health expenditure of US$63 (in US$ 2021)^32^. There are few published estimates of the costs of comprehensive adolescent preventive or screening and treatment packages for LMICs. In 2017, the Disease Control Priorities team estimated that their proposed essential packages for school-age children (school feeding, vision screening, deworming, health education and vaccines) and adolescents (media messaging on health behaviors, health education in schools and adolescent-friendly health services) would cost US$10 and US$9 per capita (in US$ 2012), respectively, in LMICs and would have high cost-effectiveness and benefit−cost^1^. However, Y-Check included a much larger number of interventions, some of which had either relatively expensive screening tests (for example, STIs) or treatment costs (for example, oral health or hearing aids). By being comprehensive, Y-Check has the potential to perform favorably when compared to the cost of multiple single-condition universal preventive interventions^33–35^. Furthermore, a scaled-up Y-Check delivered by government workers is likely to be less costly.

The effectiveness findings were largely in the anticipated direction and aligned with changes hypothesized in the intervention’s Theory of Change^15^. A novel component of the evaluation was the inclusion of educational outcomes. Our findings demonstrate the potential multisectoral impact of interventions such as Y-Check and provide supportive evidence for the need to prioritize school health interventions to improve both health and educational outcomes and to build the potential of future generations. We observed an unexpected negative impact on three self-reported behaviors. We see no plausible mechanism whereby the intervention could have led to increased sugary drink consumption, decreased condom use and poorer school results. Alternative explanations for these changes would be more truthful reporting at the follow-up visit when the participants trusted the Y-Check team and/or seasonal variation in these outcomes. Some of the changes observed between check-up and follow-up could have been due to factors external to Y-Check, although careful monitoring did not reveal any new interventions or other relevant changes. Improvements in clinical outcomes that were measured at follow-up only among those who screened positive at baseline could have been partly due to regression to the mean. Reporting bias and measurement error are possible, especially when asking adolescents about behaviors^36^ and for conditions with known measurement issues, such as blood pressure^37^. Adolescent development and maturation may have led to changes in the reporting of some outcomes—for example, intentions and self-efficacy—between baseline and follow-up.

The requirement for opt-in parental consent, concerns about confidentiality and false rumors about the intervention led to lower recruitment in secondary schools, potentially limiting the generalizability of findings from that setting. Opt-out consent, which usually leads to higher levels of participation^38^, may be an option for programmatic (non-research) implementation of Y-Check. Follow-up was lower among males and those who were out of school at the initial check-up. These limitations may have led to biases in estimates of the prevalence of conditions and behaviors and in the pre−post comparison of intervention effects on secondary outcomes but would not have impacted the primary outcome.

Findings may have limited generalizability to other health service and community contexts in Zimbabwe. For example, the availability and accessibility of referral services is likely to be lower in rural settings. However, many facilitators of successful implementation, such as the active involvement of stakeholders, and the need for enhanced support for referral uptake are likely to be generalizable. Low awareness and prioritization of non-communicable diseases and risk factors may also be common across settings.

In high-income countries, systematic reviews of the effectiveness of screening and comprehensive school-based interventions have found moderate evidence of a positive impact on health outcomes^39,40^. Although many LMICs implement routine screening programs for adolescents^14^, there are few rigorous evaluations of their implementation and effectiveness. To our knowledge, the only reported evaluation of an intervention that included multicondition screening of adolescents in Africa is a Zambian study of 380 students aged 4–16 years who attended either one of seven primary schools that had been non-randomly selected to receive the Healthy Learners Program or seven matched schools that did not^41^. The intervention was associated with lower self-reported acute illness and stunting and greater health knowledge, but there was no difference in weight or student absenteeism^41^. Our study adds to this limited body of evidence by providing evidence that Y-Check was associated with beneficial changes in multiple clinical, behavioral and educational outcomes.

The findings from this study, alongside modeling, will be used to consider whether revisions should be made to the content or delivery strategy of the Y-Check intervention in Zimbabwe, with a particular focus on maximizing the fidelity, acceptability and cost-effectiveness of the intervention. Challenges setting up and accessing appropriate referral pathways for some conditions hindered linkages between the education and health systems. Linkage to referral services is a challenge in many contexts, due to individual, service and structural factors^42,43^. We recommend providing on-site treatment services where possible and a focal person at the school or health facility to provide ongoing referral advice and support to the adolescents and their guardians. Furthermore, stronger and more integrated primary healthcare services and systems^44^ would benefit adolescents as they could receive more affordable and accessible care for multiple conditions from the same provider.

Enhanced school and community engagement is needed to support adolescents to take up the advice, treatment and care provided during Y-Check. Engagement activities should focus on building awareness of the broad range of conditions and behaviors that affect adolescents and an understanding that neglecting adolescent health now will have detrimental impacts on health, well-being and economic prosperity across the life course. The promotion of healthy lifestyles for adolescents through Y-Check should be complemented with the implementation of policies to create a supportive enabling environment. Ensuring a supportive school environment is recommended in the recently published School Nutrition Guidelines for Zimbabwe^45^, and interventions to enhance education and support for broader health and well-being in schools are outlined in the School Health Policy^46^. Implementation of these policies, in addition to broader national health and development strategies^47,48^, is needed to address the health and well-being needs of adolescents highlighted in this study and would support the sustainability of the health benefits of Y-Check.

Coordinated by the WHO, ongoing Y-Check studies will test similar adolescent check-ups that have been specifically tailored for the context in Mwanza, Tanzania, and Cape Coast, Ghana^16,17^, and will provide evidence on implementation and effectiveness in these two other urban settings^15^. In the next phase of Y-Check research, we will conduct a multicountry randomized trial to evaluate the effectiveness of the Y-Check intervention. Comprehensive health check-ups for adolescents, led by the WHO and UNICEF and informed by the Y-Check intervention, are being implemented at scale in Indonesia and Jamaica (https://www.who.int/teams/maternal-newborn-child-adolescent-health-and-ageing/adolescent-and-young-adult-health/y-check).

Our findings also provide important empirical evidence to inform upcoming WHO implementation guidance on well-care visits and to support national adoption of WHO guidance on well-care visits^13^, including recommendations on implementation features of well-care visits (such as content, timing, venue and linkages to referral care). In addition, learning from the Y-Check studies will be used to update tools such as the Universal Health Coverage Service Package Delivery and Implementation (SPDI) Tool, which supports countries to develop well-designed national health service packages (https://uhcc.who.int/uhcpackages/), and the OneHealth Tool, designed to support national strategic health priority setting using cost-effectiveness analyses (https://who.int/tools/onehealth).

In conclusion, the findings from this rigorous mixed-methods study in Zimbabwe demonstrate the potential of an intervention like Y-Check to be integrated within existing school and healthcare systems to improve the health, educational outcomes and long-term well-being of adolescents. The findings are timely and will inform upcoming WHO guidance on the implementation of check-up visits in adolescence. Future work could explore the intervention’s longer-term cost-effectiveness, sustainability and adaptability to different LMIC settings to further strengthen the case for adolescent well-care visits worldwide.

## Methods

We followed STROBE guidelines for the reporting of cohort studies (Supplementary Table 7).

### Study location

Zimbabwe was selected to participate in this study after formative research during which stakeholders indicated strong support for check-ups, as they would address an unmet need for healthcare among the adolescent population^18^. In addition, the 2018 Zimbabwean School Health Policy provides a policy framework for the provision of comprehensive school health programming^46^ and the implementation of health education and promotion. This initial Y-Check research is focusing on urban areas where there is a high number of adolescents and anticipated high levels of risk behaviors—for example, lack of exercise and substance use. Chitungwiza, with a population of 271,000, is the third-largest urban area in Zimbabwe.

### Intervention design

We developed the intervention through expert consultation, literature review, qualitative interviews and co-design workshops with adolescents and key adults in their lives, policymakers, programmers and healthcare workers^18,26^.

In 2020, formative work on the development of Y-Check in Zimbabwe involved a desk review of relevant epidemiological and health program data, participatory workshops with adolescents and parents and in-depth interviews with key informants, including policymakers, programmers and healthcare workers. The introduction of routine health check-ups was strongly supported by stakeholders, and they shared their preferences for check-up implementation format and content^18^.

In this phase of research (2021–2025), to further design the intervention content, we reviewed relevant literature and established health condition-specific technical advisory groups comprising local and international experts to advise on screening, treatment and referral protocols for each condition. Stakeholder engagement activities started in November 2021 and involved meetings with key Ministry of Health and Childcare and Ministry of Primary and Secondary Education personnel, the formation of a Youth Advisory Group and meetings with head teachers and class teachers in study schools. We also visited health facilities and met with service providers to negotiate the setup of referral pathways.

Between December 2021 and October 2022, we used a person-based design approach with adolescents (10–19 years old) to plan, test and refine the screening tools for the health and well-being check-ups^26^. Five workshops and cognitive interviewing sessions with adolescents led to modifications in the check-up content, screening tool wording and the appearance and functioning of the bespoke screening app. Additional input was received from the Youth Advisory Group and expert adolescent health and well-being stakeholders.

In June 2022, a pilot study was conducted with 171 adolescents recruited from two primary schools, two secondary schools and two community venues in Chitungwiza. After the pilot study, the check-up visit was further adapted based on the pilot screening results and process evaluation feedback from the adolescents, school staff and parents/guardians (Y-Check pilot study report available online at https://www.thruzim.org/adolescent-health-1/y-check).

### Evaluation tool design

The development of the evaluation questionnaire followed similar procedures to the intervention design and included literature review and expert consultation to identify the most appropriate questionnaire content, followed by translation into Shona and back-translation into English and pre-testing and piloting with adolescents to ensure acceptable wording and length. Existing questionnaires, such as the Global School-based Student Health Survey (https://www.who.int/teams/noncommunicable-diseases/surveillance/systems-tools/global-school-based-student-health-survey) and Demographic Health Surveys (https://www.dhsprogram.com/), informed questions on health-related knowledge, intentions and behaviors. Standard tools were used for alcohol and substance use (CRAFFT 2.1 (https://crafft.org/)), self-esteem^49^ and health-related quality of life (CHU9D)^50^. In addition, a co-design workshop with 16 adolescents explored the outcomes that are important to adolescents and led to the following youth-centered evaluation outcome: ‘Perception of the importance of getting their health checked’.

### Study design and participants

This intervention study, with a mixed-method process evaluation, was conducted in four primary schools, two secondary schools and two community venues. To align with the WHO recommendations of one check-up in early adolescence and one check-up in older adolescence^13^, the intervention was delivered to one year group in each school: Grade 5 in primary school and Form 3 in secondary school. To maximize the feasibility of the study and diversity of participants, we worked with the Ministry of Primary and Secondary Education in Chitungwiza to select schools that fulfilled the following inclusion criteria: enrollment of 200–500 Grade 5 or Form 3 students per school year, students from various socioeconomic backgrounds, and schools likely to be supportive of health interventions but with no active or recent health programs.

### Individual inclusion criteria

Primary schools: age 10–14 years, enrolled in Grade 5 in 2022, received parental consent and provided assent. Secondary schools: age 15–19 years, enrolled in Form 3 in 2022, received parental consent and provided assent. Community venues: age 16–19 years, living in a predefined catchment area near the community venue and provided their own informed consent.

### Participant recruitment and informed assent/consent

Within the selected schools, lists of potentially eligible participants were taken from school enrollment registers. Parents/guardians and students were invited to information meetings at the schools, at the end of which they were invited to sign the consent forms. They also had the option to take the forms home for further review before deciding whether to sign them. Students could participate in only the check-up visit or in both the check-up visit and the research cohort. Completed forms were returned to the study team through the schools. Prior to any study procedures, adolescents with parent/guardian consent gave their written assent. In the community venues, catchment areas surrounding each venue were clearly demarcated. Mobilizers identified adolescents aged 16–19 years who were interested in participating and accompanied them to the community venues where they were screened for eligibility and, if eligible, consented to the study.

### Procedures

The check-up took place in tents on the school grounds or at community venues and consisted of registration, an audio/computer-assisted self-completion screening questionnaire, physical assessments and nurse review (Fig. 1). Check-up data were collected on tablet computers using a bespoke, youth-friendly digital application. Y-Check staff were available to assist with tablet use and comprehension of the questions and administered the questions when necessary. In-built algorithms used the data to generate ‘warning flags’ (green, no concern; orange, some concern; red, considerable concern) for health conditions and risk behaviors. Orange and red warning flags were then reviewed by a nurse, who either discussed with or examined the adolescent to determine if further action was needed. When no issue was present, the nurse marked an orange or red warning flag as a ‘false warning flag’. Potential actions included providing information, treatment, onward referral to an in-house counselor or referral to external service providers with whom referral arrangements were negotiated in advance. Where feasible, participants were referred to government service providers; otherwise, they were referred to private for-profit or not-for-profit service providers. All adolescents received a printed screening report at the end of their visit, and those requiring referral to an external service provider were provided with a paper referral form. The issues screened for and potential actions taken are described in Supplementary Table 4. The nurse also applied fluoride gel to all participants’ teeth to prevent cavities and provided general health information and advice, including proper tooth and gum brushing techniques, good sleep habits, physical activity and maintaining a healthy balanced diet. Physical games were available to the adolescents while they were waiting, and all participants received a healthy food snack, toothbrush and toothpaste. In addition, females received reusable menstrual hygiene pads, two pairs of underwear, a bar of soap and information on menstrual health.

Attendance at the first referral appointment was recorded through a paper referral form that the participant took to the referral appointment. Prior to the start of the intervention, service providers were orientated to the Y-Check study and completion of the paper referral form. Memoranda of understanding (MoU) were signed with providers that outlined the services that would be provided and any associated costs. Incidental findings not included in the MoU would not be covered by the study. The check-up visit and all clinical costs related to the first referral services were provided free of charge to the participants as long as this occurred within 6 months of the check-up. However, where the participant had been non-contactable, their referral costs were covered if they were then contacted during the follow-up visit and attended the referral appointment (up to 10 months after check-up). Some referral facilities were in Chitungwiza, but other services were available only in Harare, 25 km away. Group transport for adolescents and their parent or guardian was provided for referrals to Harare but not within Chitungwiza.

### Confidentiality and information sharing

All medical information was deidentified and recorded using a unique study identification number. Identifying information, such as names, addresses and phone numbers, was recorded in a registration book that was stored in a locked container when not in use. Adolescents received their screening results verbally from the nurse. They also received a letter with results for them to take home to their parent/guardian. Medical information was not shared with other students or teachers. However, in instances where a participant was identified as being suicidal and required emergency care, the school health coordinator and school head teacher were informed. Decisions around who would accompany the young person to the referral service took place in consultation with school staff, parents/guardians and/or trusted adults who had been nominated by older adolescents at community venues during registration.

### Outcome evaluation

At baseline, just prior to the health check-up, data on sociodemographic variables and some of the evaluation outcomes were collected using an adolescent self-completion structured questionnaire programmed in Open Data Kit (ODK). Follow-up questionnaires and measurements were conducted 5–7 months after intervention. The follow-ups were done at the same schools and community venues, with participants invited through schools and via phone calls by the community mobilizers. If the participant’s phone number was unreachable, SMS messages were sent requesting that the participant get in touch as soon as they received the message. Where participants were unreachable over the phone, up to three home visit attempts were made before determining them as lost to follow-up. Participants who had relocated to other parts of the country were offered bus fare reimbursement for them to return for the follow-up visit.

The primary outcome was the proportion of participants who screened positive for at least one issue (condition or behavior) and received appropriate on-the-spot care and/or completed appropriate referrals for all identified issues by the time of the follow-up. The list of health issues included in the primary outcome is provided in Supplementary Table 4. Completing appropriate referral was defined as attending at least one referral visit for each issue identified, which was measured by retrieving the referral form from the service provider. Participants who were already receiving care for their issue(s) and who did not require further intervention were considered to have received appropriate on-the-spot care. Secondary implementation outcomes included the proportion of participants who screened positive for individual issues and received appropriate on-the-spot care and/or completed appropriate referrals by the time of the follow-up visit as well as the yield of untreated conditions and the prevalence of risk behaviors. We also evaluated the status at follow-up of the following previously diagnosed conditions: STI symptoms, STI test positive, symptoms suggestive of depression and/or anxiety, suicide risk, epilepsy and visual and hearing impairment (Supplementary Table 5).

Other implementation outcomes were assessed through a mixed-methods process evaluation, guided by the Medical Research Council process evaluation framework^51^. We adapted Proctor’s Implementation Outcomes Framework^52^ to focus on five of the implementation outcomes: acceptability, adoption, appropriateness, feasibility and fidelity.

Secondary individual outcomes, mapped onto the Theory of Change^15^, were health-related knowledge (measured using an six/eight-item quiz); intention to adopt a healthy behavior (avoid drugs and alcohol and be more active); agency to make decisions about health; health-related risk and protective behaviors (sweetened drink consumption, fruit consumption, support for mental health, sleep, physical activity, substance and alcohol use, smoking, tooth brushing, sexual risk behavior and HIV testing); engagement with health services in the past 4 months; self-esteem (Rosenberg self-esteem scale); health-related quality of life (CHU9D) and life satisfaction as a measure of subjective well-being; clinical outcomes (anemia, thinness (body mass index (BMI) for age and sex (*z*-score less than −2 s.d.)) and obesity (BMI for age and sex (*z*-score greater than +2 s.d.)); educational outcomes (days missed in the past month due to illness or menstruation, perception of school results, impaired performance or reduced participation due to ill-health); and perception of the importance of getting health checked (youth-centered outcome) (Supplementary Table 5).

### Sample size

The sample size calculations assumed that 30% of participants would screen positive for at least one issue (condition/behavior) and that 75% of those who screened positive would be correctly managed. With 500 participants in total, and 150 screening positive for at least one issue in each of the four age/sex groups, the 95% confidence interval for the primary outcome of correct management would be 68.0−82.0%.

### Process evaluation

Process evaluation data were collected using diverse qualitative and quantitative methods (Supplementary Table 6). Qualitative data were generated by S.M., F.R.K., C.A.N. and C.R.S.M.-Y., who were distinct from the intervention team but collaborated closely with them throughout.

#### Observations

Non-participant observations were conducted of (1) the Y-Check screening, (2) referral services provision and (3) intervention team biweekly debrief meetings. All observations were conducted with the awareness of participants and service providers. Observations of screening and referral services centered on interactions of intervention staff, adolescents and service providers during the provision and receiving of services. Informal conversations with intervention staff, participants and service providers were also conducted during observation visits, and, in some cases, data generated during observations were explored in more detail within in-depth interviews. Observations of biweekly team debriefing meetings were conducted to understand experiences, challenges and adaptations in intervention delivery. Observations were guided by semi-structured observation guides. Detailed field notes and minutes were captured during observations and written up after each observation. The observation notes for each observation included a section on reflection, which included specific reflections on ‘How do you think your presence influenced what happened during the observation?’

#### In-depth interviews

Qualitative interviews were conducted with adolescents who received the Y-Check intervention (*n* = 9), adolescents who were referred (*n* = 10), service providers (*n* = 5), intervention staff (*n* = 7) and school authorities (*n* = 6) using the participants’ preferred language (English or Shona). A range of participants was purposively selected, based on the following characteristics: location of intervention (that is, primary school, secondary school or community), sex and age. In-depth interviews were guided by topic guides that explored the acceptability, appropriateness, feasibility, fidelity and adoption of the Y-Check intervention. All interviews were conducted in a private setting (at school or in a participantʼs home), were audio recorded, transcribed and, when necessary, translated into English and lasted around 45–60 minutes.

#### Participatory workshops

Thirteen participatory workshops (8–10 participants) were held separately with adolescents, their parents and teachers within 3 months of the initial check-up. These included adolescents who participated in the Y-Check intervention (*n* = 5 workshops), adolescents who did not participate in the intervention (*n* = 4 workshops), parents of adolescents who participated in the Y-Check intervention (*n* = 2 workshops) and parents of adolescents who did not participate in the intervention (*n* = 2 workshops). All participatory workshops were guided by semi-structured guides, covering topics around (non)acceptability, appropriateness, feasibility and sustainability of the intervention. Additionally, workshops with parents covered topics on how parental support and consent might be improved. All workshops were facilitated in the participants’ preferred language (English or Shona), were recorded and lasted around 2−3 hours each.

There was only one major deviation from the published protocol^15^. The original design proposed a first follow-up visit after 4 months with a second follow-up at 12 months^15^, but, due to implementation delays, the first follow-up visits occurred 5–7 months after the intervention. As a result, the planned second follow-up at 12 months did not take place as it was considered too close to the first follow-up to provide substantial additional value.

### Cost analysis

We followed the International Decision Support Initiative reference case to guide the planning, conduct and reporting of the economic evaluation of Y-Check (https://idsihealth.org/resource-items/idsi-reference-case-for-economic-evaluation/). In this paper, we report analysis from the provider’s perspective. The intervention ran for 18 months and included a start-up phase (intervention development and piloting, from September 2021 to September 2022) and an implementation phase (from October 2022 to March 2023). Costs associated with referrals to external service providers that took place between October 2022 and September 2023 were included.

A combination of top-down and ingredients-based costing approaches was used to identify, measure and value resources used for delivering the whole package and for each component/activity. Resources used were identified and measured using process evaluation data, document review and financial and accounting records. Staff time spent on each service (that is, time spent testing or treating per condition/risk behavior) was collected through interviews and ODK timestamps. Both financial and economic costs were considered. All research costs, such as monitoring and evaluation, were excluded. Expenses were incurred either in US$ or the now-obsolete Zimbabwean Dollar (ZWL). All costs paid in ZWL were converted to US$ at the spot rate of the date of the transaction. Costs are presented in US$ 2023 and are discounted at 3% as the international standard.

The Y-Check intervention costs were analyzed using an adapted Excel-based costing tool^53^. We estimated the total cost of setting up and implementing the Y-Check intervention in school and community settings, including testing, on-the-spot care, referral and administrative/management costs. To understand the costs around implementing the check-up itself, we also estimated:

1. The cost per adolescent reached, which was calculated as the total cost of implementing the Y-Check intervention (that is, screening and treating the participants), excluding referral costs and indirect or support costs (for example, administrative or support activities not directly associated with delivery), divided by the total number of adolescents who received Y-Check.
2. The total cost to screen, treat and/or refer participants, which was calculated as the total cost of implementing Y-Check, excluding indirect or support costs (for example, administrative or support activities not directly associated with delivery), divided by the number of adolescents who received Y-Check.

### Statistical analysis

Analysis was conducted in Stata 18. The proportion of potentially eligible adolescents aged 16–17 years in community settings was estimated using population census data. Participants’ baseline sociodemographic characteristics were used to compare the community-level and school-level characteristics of study communities and schools. Sociodemographic characteristics of participants at baseline and follow-up were compared.

The screening test results, services delivered on the spot and referrals made and completed were collated and reported by sex and age group and/or location. Survey implementation variables were described at each timepoint.

The primary outcome and secondary outcomes measured at a single timepoint were estimated as a proportion with a 95% confidence interval for four age/sex groups: 10−14-year-old males, 10–14-year-old females, 15–19-year-old males and 15–19-year-old females. Preplanned sensitivity analyses for the primary outcome included (1) adjustments to the definition of ‘screen positive’ to account for incorrect app algorithms; (2) limiting the outcome to receipt of on-the-spot care only; (3) using self-reports as well as documented attendance as evidence of attendance at referral appointments; and (4) restricting referral attendance to only those who attended within 4 months. Two additional post hoc sensitivity analyses were conducted: (1) restricting the analysis to issues applicable to all sex and age groups and (2) excluding issues that were previously diagnosed and did not require further management.

For conditions where follow-up clinical outcomes were measured only among those who screened positive at baseline, the prevalence of the condition at follow-up was calculated, with a 95% confidence interval. For outcomes that were measured at two timepoints, a pre−post analysis was conducted to compare differences in measures between the two timepoints. The percentage difference in prevalence of each outcome was calculated with 95% confidence intervals. Prevalence ratios and 95% confidence intervals were estimated using a mixed-effects population-averaged generalized linear model with Bernoulli distribution and logit link function, adjusting for school/community as a fixed effect. The Wald test was used to estimate *P* values.

The proportion of missing values for individual conditions was reported as a percentage of those who were eligible to be assessed for the condition. Baseline information from clients lost to follow-up was retained in relevant analyses.

### Qualitative analysis

Analysis of the implementation of Y-Check was guided by Proctor’s Implementation Outcomes Framework^52^, focusing on the five implementation outcome domains that are most relevant to this phase of Y-Check research (that is, Feasibility, Fidelity, Adoption, Acceptability and Appropriateness). The qualitative and quantitative data that were relevant to each domain were identified from the full dataset. Qualitative data were manually analyzed thematically and inductively. We composed an analytical memo for each domain and then subsequently refined this iteratively by triangulating with data across different sources and grouping the data thematically. This process of analytical refinement was guided by regular discussions within the process evaluation team to discuss key emerging sub-themes.

Further details on the intervention and its evaluation are provided in the published study protocol^15^.

### Inclusion and ethics statement

This study was designed through a longstanding partnership among the Biomedical Research and Training Institute (BRTI) in Harare, Zimbabwe, the Zimbabwean Ministries of Health and Childcare (MoHCC) and Primary and Secondary Education (MoPSE), the London School of Hygiene & Tropical Medicine (LSHTM) and the WHO, which has led to many collaborations, including research studies on young people’s health^18,54^, capacity strengthening (Southern Africa Research Capacity Network, https://www.sofarafrica.org/our-programme) and professional development^55^. Chitungwiza was selected by the BRTI as a study setting owing to its proximity to the research center in Harare and a long-established collaboration with Chitungwiza City Health. Formative work conducted in Chitungwiza in 2020 (ref. ^18^) and previous work with young people informed the study procedures and ethical considerations.

In keeping our focus on designing contextually relevant interventions, intervention and evaluation procedures were designed with Zimbabwean investigators and youth researchers, with regular input from an Adolescent Advisory Group from the study community and external expert advisors (national and international). Discussions with MoHCC and MoPSE and previous work from this region were used to guide the design of the study and have been taken into account in the citations for this paper. The study was coordinated by Zimbabwean researchers, and team members collaborated on data ownership, intellectual property and authorship of publications related to the work. Roles and responsibilities were agreed upon among researchers ahead of the research. The principal investigator (A.M.D.) and LSHTM researchers (V.S., R.A.F. and C.R.S.M.-Y.) were based full-time in Zimbabwe during the study implementation and provided technical support to the Zimbabwean study coordinator and to field and data teams. Two team members embedded their doctoral research within the Y-Check study. In addition, three master’s degrees, two bachelorʼs degrees and two diploma courses for Zimbabwean staff were supported within the Y-Check study.

Providers were specifically trained on how to communicate with and address the specific needs of LGBT+ clients. Where feasible, the needs of people with disabilities were addressed—for example, by providing support when completing questionnaires and adapting screening procedures. Standard operating procedures and training ensured that providers were safe—for example, post-exposure prophylaxis, safe lifting and handling procedures and keeping safe after hours. The study started toward the end of the COVID-19 pandemic, and personal protective equipment was provided to staff and participants, and staff were trained on infection prevention and control procedures.

### Ethics approval

Ethics approval was granted by the institutional review boards of the Medical Research Council of Zimbabwe (MRCZ/A/2766), the institutional ethics committee of the Chitungwiza City Health Department, the LSHTM (26395) and the WHO (ERC.0003778). Primary school students aged 10–14 years and secondary school students aged 15–19 years could participate in the study if their parent/guardian consented and they also provided their assent. At community venues, adolescents aged 16–19 years provided their own informed consent. The study protocol and statistical analysis plan are available at 10.17037/DATA.00004763. The study is also registered at ClinicalTrials.gov: NCT06090006.

### Reporting summary

Further information on research design is available in the Nature Portfolio Reporting Summary linked to this article.

## Online content

Any methods, additional references, Nature Portfolio reporting summaries, source data, extended data, supplementary information, acknowledgements, peer review information; details of author contributions and competing interests; and statements of data and code availability are available at 10.1038/s41591-025-04156-x.

## Supplementary information

Supplementary Information Supplementary Tables 1–7.

Reporting Summary

## Figures and Tables

**Extended Data Table 1:**
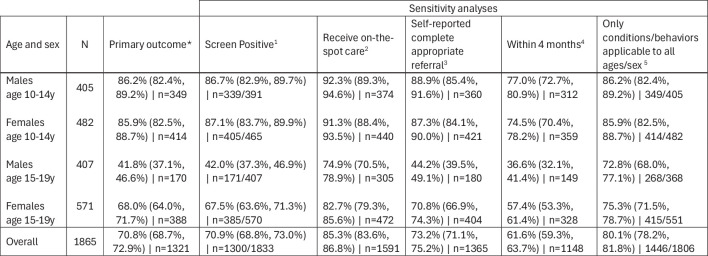
Primary outcome (95% CI) distributed by age and sex and related sensitivity analysis

* Primary outcome was defined as the proportion of participants screening positive for at least one issue (condition or behavior) who received appropriate on-the-spot care and/or completed appropriate referral, with documented attendance, for all screened issues, by the time of follow-up visit.

Sensitivity analyses: ^1^ Post-implementation identification of red warning flags following the screening protocol. The denominator of the outcome in the sensitivity analysis differs from the primary outcome as it includes warning flags that may not have been defined during data collection due to implementation errors and excludes erroneous flags (false positives) defined as red warning flags during implementation. ^2^ If a warning flag required both on-the-spot care and referral, the outcome will be based only on whether the participant received appropriate on-the-spot care, regardless of their referral attendance. Those who refused treatment/referral and/or those who refused discussion on the topic are considered as having received appropriate on-the-spot care. ^3^ Completed appropriate referral by referral form retrieved from the service provider (documented) or self-reported referral attendance during follow-up visit. ^4^ Completed referral (documented attendance) within 4 months of check-up visit. ^5^ Analysis restricted to issues applicable to all ages and sex, by removing sexual and reproductive health (sexual risk, contraception, STIs, HIV and VMMC) and blood pressure.

**Extended Data Table 2:**
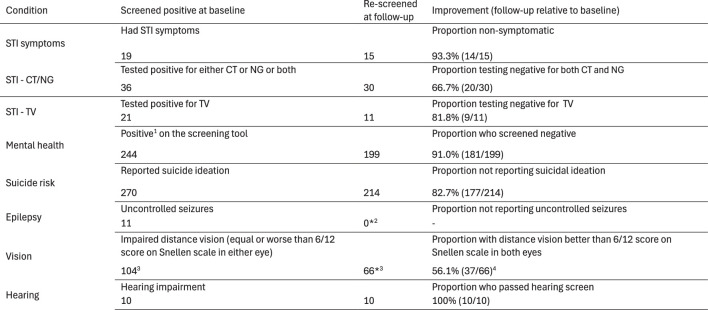
Clinical outcomes for those who screened positive and had repeat assessment at follow-up

STI, sexually transmitted infections; CT, *Chlamydia trachomatis*; NG, *Neisseria gonorrhoeae*; TV, *Trichomonas vaginalis*.

^1^ Positive was ≥10 for the Patient Health Questionnaire-9, ≥8 for the General Anxiety Disorder-7 and ≥15 for the Pediatric Symptom Checklist-17.

^2^ Frequency of seizures was not asked at follow-up.

^3^ In total, 150 were flagged during implementation. Based on the rescreening scores at follow-up, 46 were excluded because they had normal vision without correction at the follow-up visit (false flags), leaving 104 screen positive for vision impairment at baseline.

^3^ In total, 128 of 150 were seen at follow-up, of whom 112 (87.5%) were rescreened at follow-up. Based on the rescreening scores, 46 were excluded because they had normal vision without correction at the follow-up visit (false flags), leaving 66. These 66 were used as the denominator for outcome improvement analysis.

^4^ Further restricting the analysis to those who received spectacles and excluding one participant with a missing measurement, the denominator is 51. In total, 14 of 51 remained with visual impairment with their spectacles on at follow-up (27.45%), whereas 37 (72.55%) had visual acuity of better than 6/12 at follow-up.

**Extended Data Table 3:**
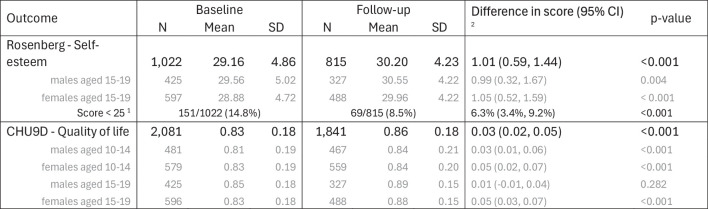
Pre−post comparison of mean scores of RSES and CHU9D

^1^ Low self-esteem score below 25, presenting proportion at baseline and at follow-up and the difference in proportion with a two-sample test of proportions.

^2^We conducted a mixed-effects generalized linear model fitting population-averaged panel data, adjusting for the random effects of location. RSES, Rosenberg self-esteem scale; CHU9D, Child Health Utility 9D.

**Extended Data Table 4:**
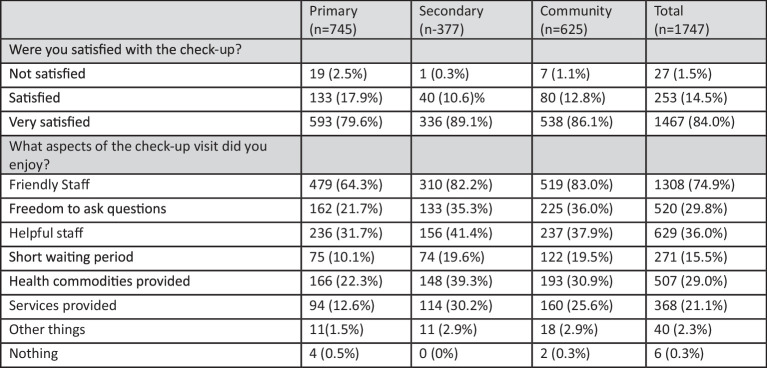
Level of satisfaction with the check-up visit as reported during exit interviews

## Data Availability

Data analyzed in this paper were collected with an ethical commitment that they would be accessed by authorized users and used for study purposes only. Requests for data should be sent to the corresponding author, A.M.D., or by completing a request form at 10.17037/DATA.00004763. Requests will be considered by the Y-Check study management group, which includes the principal investigator, data manager, statistician and study coordinator. Responses to requests for data will be provided within 2 weeks and will be communicated by the corresponding author.

## Code availability

Processing code and documentation, including the study protocol and the manual of operations, have been made openly available under a Creative Commons license at 10.17037/DATA.00004763.
