# Supplementary material for: Implementation and evaluation of the Y-Check comprehensive adolescent health check-up intervention in Zimbabwe: a pre−post mixed-methods study
Source: Nat Med. 2026 Feb 2;32(2):494–504. doi: 10.1038/s41591-025-04156-x (PMC12920085; doi:10.1038/s41591-025-04156-x)
Supplement: Supplementary file 1 — Supplementary Tables 1–7. [file 41591_2025_4156_MOESM1_ESM.pdf]

nature medicine

Supplementary information https://doi.org/10.1038/s41591-025-04156-x

# Implementation and evaluation of the Y-Check comprehensive adolescent health check-up intervention in Zimbabwe: a pre–post mixed-methods study

In the format provided by the authors and unedited

***Supplementary material***

***Supplementary Table 1 - Proportion of cohort followed-up according to baseline socio-demographic characteristics***

| | | Primary | | Secondary | | Community | |
|---|---|---|---|---|---|---|---|
| **Variable** | **Categories** | **Baseline (n=1071)** | **Proportion followed up (n=1028)** | **Baseline (n=387)** | **Proportion followed up (n=351)** | **Baseline (n=639)** | **Proportion followed up (n=464)** |
| **Total** | | | **96.0%** | | **90.7%** | | **72.6%** |
| **Age at baseline** | **10-11y** | 844 | 816 (96.7%) | | | | |
| | **12-14y** | 227 | 212 (93.4%) | | | | |
| | **15-16y** | | | 271 | 246 (90.8%) | 287 | 215 (74.9%) |
| | **17-19y** | | | 116 | 105 (90.5%) | 352 | 249 (70.7%) |
| **Sex** | **Male** | 485 | 467 (96.3%) | 140 | 129 (92.1%) | 287 | 198 (69.0%) |
| | **Female** | 586 | 561 (95.7%) | 247 | 222 (89.9%) | 352 | 266 (75.6%) |
| **In-school at baseline** | **In-school** | | | | | 484 | 358 (74.0%) |
| | **Out of school** | | | | | 155 | 106 (68.4%) |
| **Asset index at baseline** | **1st quintile (lowest)** | 162 | 154 (95.1%) | 65 | 57 (87.7%) | 133 | 103 (77.4%) |
| | **2nd** | 165 | 157 (95.2%) | 78 | 70 (89.7%) | 120 | 84 (70.0%) |
| | **3rd** | 169 | 165 (97.6%) | 84 | 80 (95.2%) | 118 | 80 (67.8%) |
| | **4th** | 181 | 173 (95.6%) | 69 | 60 (87.0%) | 110 | 87 (79.1%) |
| | **5th quintile (highest)** | 190 | 184 (96.8%) | 55 | 50 (90.9%) | 94 | 67 (71.3%) |
| | ***Missing*** | 204 | 195 (95.6%) | *36* | 34 (94.4%) | *64* | 43 (67.2%) |

*Supplementary Table 2 - Proportion of participants screening positive who received appropriate on-the-spot care and/or completed appropriate referral by condition/behavior (Secondary Outcome)*

| Condition / Behaviour | Proportion of participants screening positive for each condition who received appropriate on-the-spot care or completed appropriate referral (documented attendance) by the time of follow-up | | | | |
|---|---|---|---|---|---|
| | Ages 10-14 | | Ages 15-19 | | Total |
| | Males | Females | Males | Females | |
| **Psychosocial issues & CMDs & suicide risk** | 200/204 (98.0%) | 229/235 (97.4%) | 183/208 (88.0%) | 271/330 (82.1%) | 883/977 (90.4%) |
| **Substance use/smoking** | 43/43 (100.0%) | 20/21 (95.2%) | 173/178 (97.2%) | 138/143 (96.5%) | 374/385 (97.1%) |
| **Epilepsy** | 2/2 (100.0%) | 1/5 (20.0%) | 0/0 | 2/4 (50.0%) | 5/11 (45.5%) |
| **Hearing impairment** | 2/2 (100.0%) | 2/3 (66.7%) | 4/4 (100.0%) | 1/1 (100.0%) | 9/10 (90.0%) |
| **Vision impairment** | 20/26 (76.9%) | 29/37 (78.4%) | 24/33 (72.7%) | 43/54 (79.6%) | 116/150 (77.3%) |
| **Oral health condition** | 30/45 (66.7%) | 45/56 (80.4%) | 25/56 (44.6%) | 37/80 (46.2%) | 137/237 (57.8%) |
| **Physical impairment** | 3/3 (100.0%) | 6/8 (75.0%) | 10/14 (71.4%) | 4/7 (57.1%) | 23/32 (71.9%) |
| **Malnutrition** | 12/47 (25.5%) | 19/59 (32.2%) | 1/33 (3.0%) | 22/48 (45.8%) | 54/187 (28.9%) |
| **Anaemia** | 30/30 (100.0%) | 56/56 (100.0%) | 37/38 (97.4%) | 120/123 (97.6%) | 243/247 (98.4%) |
| **VMMC need** | N/A | N/A | 0/145 (0.0%) | N/A | 0/145 (0.0%) |
| **HIV** | N/A | N/A | 2/4 (50.0%) | 8/10 (80.0%) | 10/14 (71.4%) |
| **Family planning need** | N/A | N/A | 35/36 (97.2%) | 46/46 (100.0%) | 81/82 (98.8%) |
| **STIs** | N/A | N/A | 7/10 (70.0%) | 44/56 (78.6%) | 51/66 (77.3%) |
| **Elevated blood pressure** | N/A | N/A | 92/140 (65.7%) | 128/179 (71.5%) | 220/319 (69.0%) |
| **Oral hygiene** | 178/178 (100.0%) | 196/198 (99.0%) | 139/139 (100.0%) | 135/135 (100.0%) | 648/650 (99.7%) |
| **Poor sleep** | 36/36 (100.0%) | 35/35 (100.0%) | 72/72 (100.0%) | 143/143 (100.0%) | 286/286 (100.0%) |
| **Limited physical activity** | 208/208 (100.0%) | 261/261 (100.0%) | 133/133 (100.0%) | 360/360 (100.0%) | 962/962 (100.0%) |
| **Sexual risk** | N/A | N/A | 49/50 (98.0%) | 37/37 (100.0%) | 86/87 (98.9%) |
| **Schistosomiasis** | 12/12 (100.0%) | 6/7 (85.7%) | 15/22 (68.2%) | 5/8 (62.5%) | 38/49 (77.6%) |

*Key: CMDs Common mental disorders; VMMC Voluntary Medical Male Circumcision; STIs Sexually Transmitted Infections*

*Supplementary Table 3a: Before-after comparison of prevalence of specific conditions/behaviors (males aged 10-14)*

| | Baseline n/N (%) | Follow-up n/N (%) | Difference in proportions (95% CI) | PR (95% CI) | p-value |
|---|---|---|---|---|---|
| **Health knowledge quiz scores >= 3/6** | 92/482 (19.1) | 107/465 (23.0) | 3.9 (-1.3, 9.1) | 1.20 (0.97, 1.49) | 0.100 |
| **Has sugary drink less than once per day** | 377/482 (78.2) | 312/465 (67.1) | -11.1 (-16.7, -5.5) | 0.85 (0.79, 0.92) | 0.000 |
| **Eats fruit at least once per day** | 119/482 (24.7) | 144/465 (31.0) | 6.3 (0.6, 12.0) | 1.24 (1.00, 1.52) | 0.045 |
| **Brushes teeth at least once a day with toothpaste** | 353/485 (72.8) | 359/464 (77.4) | 4.6 (-0.9, 10.1) | 1.07 (1.01, 1.14) | 0.025 |
| **Sleeps at least 8 hours** | 436/485 (89.9) | 419/465 (90.1) | 0.2 (-3.6, 4.0) | 1.01 (0.97, 1.05) | 0.778 |
| **Physically active every/almost every day in past 2 weeks** | 350/485 (72.2) | 347/465 (74.6) | 2.4 (-3.2, 8.0) | 1.05 (0.98, 1.12) | 0.188 |
| **Low or no substance use (CRAFFT score <2)** | 465/485 (95.9) | 449/465 (96.6) | 0.7 (-1.7, 3.1) | 1.00 (0.98, 1.02) | 0.690 |
| **No smoking in past 30 days** | 481/485 (99.2) | 458/465 (98.5) | -0.7 (-2.1, 0.7) | 0.99 (0.97, 1.00) | 0.080 |
| **Has support with mental health challenges** | 89/104 (85.6) | 102/117 (87.2) | 1.6 (-7.5, 10.7) | 1.00 (0.92, 1.10) | 0.924 |
| **Attended clinic in past 4 months [if sick and wanted to go]** | 41/63 (65.1) | 55/135 (40.7) | -24.4 (-38.8, -10.0) | 0.61 (0.47, 0.80) | 0.000 |
| **Satisfied with life [5 stars]** | 317/482 (65.8) | 312/467 (66.8) | 1.0 (-5.0, 7.0) | 1.01 (0.93, 1.10) | 0.818 |
| **Not thin/severely thin** | 454/485 (93.6) | 447/466 (95.9) | 2.3 (-0.5, 5.1) | 1.02 (1.00, 1.04) | 0.021 |
| **Not obese** | 474/485 (97.7) | 455/466 (97.6) | -0.1 (-2.0, 1.8) | 1.00 (0.99, 1.01) | 0.643 |
| **No anaemia** | 451/480 (94.0) | 431/465 (92.7) | -1.3 (-4.5, 1.9) | 0.99 (0.96, 1.02) | 0.538 |
| **No severe anaemia** | 480/480 (100.0) | 465/465 (100.0) | 0.0 (0.0, 0.0) | - | - |
| **No school missed due to illness in the past month** | 242/481 (50.3) | 265/467 (56.7) | 6.4 (0.1, 12.7) | 1.12 (1.01, 1.24) | 0.025 |
| **School results OK/good/excellent** | 437/473 (92.4) | 411/465 (88.4) | -4.0 (-7.8, -0.2) | 0.95 (0.92, 0.98) | 0.003 |
| **Health did not impair school performance in past 4 months** | 281/482 (58.3) | 317/467 (67.9) | 9.6 (3.5, 15.7) | 1.17 (1.08, 1.27) | 0.000 |
| **Health did not impair school participation in past 4 months** | 312/482 (64.7) | 328/467 (70.2) | 5.5 (-0.5, 11.5) | 1.09 (1.01, 1.17) | 0.031 |
| **Health checks are worthwhile** | 284/482 (58.9) | 323/467 (69.2) | 10.3 (4.2, 16.4) | 1.16 (1.06, 1.26) | 0.001 |

See Supplementary Table 5 for secondary outcomes definitions

*[1] Prevalence ratios derived from population-averaged generalised linear models adjusted for within-location correlation.

(-) Prevalence ratio and respective p-value not calculated for secondary outcomes with data sparsity (log-binomial model did not converge)

*Supplementary Table 3b: before-and-after comparison of prevalence of specific conditions/behaviors (male aged 15-19)*

| | Baseline n/N (%) | Follow-up n/N (%) | Difference in proportions (95% CI) | PR (95% CI) | p-value |
|---|---|---|---|---|---|
| **Health knowledge quiz scores >= 4/8** | 108/426 (25.4) | 71/327 (21.7) | -3.7 (-9.8, 2.4) | 0.85 (0.67, 1.07) | 0.171 |
| **Strong desire to avoid drugs/alcohol** | 354/426 (83.1) | 280/327 (85.6) | 2.5 (-2.7, 7.7) | 1.04 (0.98, 1.09) | 0.212 |
| **Strong desire to be active** | 365/426 (85.7) | 291/327 (89.0) | 3.3 (-1.4, 8.0) | 1.04 (0.99, 1.09) | 0.098 |
| **Empowered to make health-related decisions** | 150/426 (35.2) | 143/327 (43.7) | 8.5 (1.5, 15.5) | 1.30 (1.12, 1.52) | 0.001 |
| **Has sugary drink less than once per day** | 360/426 (84.5) | 237/327 (72.5) | -12.0 (-17.9, -6.1) | 0.86 (0.80, 0.92) | 0.000 |
| **Eats fruit at least once per day** | 105/426 (24.6) | 91/327 (27.8) | 3.2 (-3.1, 9.5) | 1.12 (0.89, 1.40) | 0.339 |
| **Brushes teeth with toothpaste at last once per day** | 358/426 (84.0) | 291/327 (89.0) | 5.0 (0.1, 9.9) | 1.06 (1.01, 1.11) | 0.024 |
| **Sleeps at least 8 hours** | 348/426 (81.7) | 234/327 (71.6) | -10.1 (-16.2, -4.0) | 0.89 (0.83, 0.96) | 0.003 |
| **Physically active every/almost every day in past 2 weeks** | 292/426 (68.5) | 231/327 (70.6) | 2.1 (-4.5, 8.7) | 1.05 (0.96, 1.14) | 0.265 |
| **Low or no substance use (CRAFFT score <2)** | 348/426 (81.7) | 298/327 (91.1) | 9.4 (4.6, 14.2) | 1.12 (1.07, 1.17) | 0.000 |
| **No smoking in past 30 days** | 378/426 (88.7) | 312/323 (96.6) | 7.9 (4.3, 11.5) | - | - |
| **Has support with mental health challenges** | 44/51 (86.3) | 42/47 (89.4) | 3.1 (-9.8, 16.0) | 1.11 (0.98, 1.25) | 0.090 |
| **Had <=1 sexual partner in past 6 months** | 244/276 (88.4) | 177/198 (89.4) | 1.0 (-4.7, 6.7) | 1.01 (0.96, 1.07) | 0.612 |
| **Uses condoms most/all the time** | 43/65 (66.2) | 9/53 (17.0) | -49.2 (-64.5, -33.9) | 0.25 (0.13, 0.47) | 0.000 |
| **Using effective contraception** | 51/70 (72.9) | N/A | - | - | - |
| **Linked to care if HIV+** | 10/15 (66.7) | 10/17 (58.8) | -7.9 (-41.3, 25.5) | - | - |
| **Attended clinic in past 4 months [if sick and wanted to go]** | 13/28 (46.4) | 11/30 (36.7) | -9.7 (-35.0, 15.6) | 0.87 (0.56, 1.34) | 0.520 |
| **Satisfied with life [5 stars]** | 182/426 (42.7) | 147/327 (45.0) | 2.3 (-4.9, 9.5) | 1.06 (0.94, 1.21) | 0.343 |
| **Not thin/severely thin** | 387/425 (91.1) | 299/319 (93.7) | 2.6 (-1.2, 6.4) | 1.03 (1.01, 1.06) | 0.005 |
| **Not obese** | 422/425 (99.3) | 316/319 (99.1) | -0.2 (-1.5, 1.1) | - | - |
| **No anaemia** | 383/421 (91.0) | 308/327 (94.2) | 3.2 (-0.5, 6.9) | 1.03 (1.00, 1.07) | 0.047 |
| **No severe anaemia** | 421/421 (100.0) | 327/327 (100.0) | 0.0 (0.0, 0.0) | - | - |
| **No school/work missed due to illness in the past month** | 248/379 (65.4) | 199/293 (67.9) | 2.5 (-4.7, 9.7) | 1.04 (0.95, 1.14) | 0.429 |
| **School results OK/good/excellent** | 308/376 (81.9) | 219/272 (80.5) | -1.4 (-7.5, 4.7) | 0.99 (0.93, 1.06) | 0.785 |
| **Health did not impair school/work performance in past 4 months** | 219/426 (51.4) | 188/327 (57.5) | 6.1 (-1.1, 13.3) | 1.14 (1.03, 1.26) | 0.015 |
| **Health did not impair school/work participation in past 4 months** | 259/426 (60.8) | 211/327 (64.5) | 3.7 (-3.3, 10.7) | 1.08 (0.99, 1.18) | 0.079 |
| **Health checks are worthwhile** | 292/426 (68.5) | 232/327 (70.9) | 2.4 (-4.2, 9.0) | 1.03 (0.95, 1.12) | 0.498 |

See Supplementary Table 5 for secondary outcomes definitions

*[1] Prevalence ratios derived from population-averaged generalised linear models adjusted for within-location correlation.

(-) Prevalence ratio and respective p-value not calculated for secondary outcomes with sparse data (log-binomial model did not converge)

*[2] Effective contraception not screened for at follow-up for males.

*Supplementary Table 3c: before-and-after comparison of prevalence of specific conditions/behaviors (female aged 10-14)*

| | Baseline n/N (%) | Follow-up n/N (%) | Difference in proportions (95% CI) | PR (95% CI) | p-value |
|---|---|---|---|---|---|
| **Health knowledge quiz scores >= 3/6** | 119/581 (20.5) | 126/559 (22.5) | 2.0 (-2.8, 6.8) | 1.08 (0.88, 1.33) | 0.455 |
| **Has sugary drink less than once per day** | 436/581 (75.0) | 382/559 (68.3) | -6.7 (-11.9, -1.5) | 0.91 (0.85, 0.98) | 0.011 |
| **Eats fruit at least once per day** | 143/581 (24.6) | 167/559 (29.9) | 5.3 (0.1, 10.5) | 1.22 (1.02, 1.46) | 0.029 |
| **Brushes teeth at least once a day with toothpaste** | 454/584 (77.7) | 468/558 (83.9) | 6.2 (1.7, 10.7) | 1.08 (1.03, 1.12) | 0.001 |
| **Sleeps at least 8 hours** | 537/584 (92.0) | 523/559 (93.6) | 1.6 (-1.4, 4.6) | 1.01 (0.98, 1.05) | 0.347 |
| **Physically active every/almost every day in past 2 weeks** | 387/584 (66.3) | 369/559 (66.0) | -0.3 (-5.8, 5.2) | 0.99 (0.92, 1.06) | 0.699 |
| **Low or no substance use (CRAFFT score <2)** | 573/584 (98.1) | 553/559 (98.9) | 0.8 (-0.6, 2.2) | - | - |
| **No smoking in past 30 days** | 582/584 (99.7) | 558/558 (100.0) | 0.3 (-0.1, 0.7) | - | - |
| **Has support with mental health challenges** | 80/97 (82.5) | 83/101 (82.2) | -0.3 (-10.9, 10.3) | 0.99 (0.88, 1.12) | 0.903 |
| **Attended clinic in past 4 months [if sick and wanted to go]** | 50/75 (66.7) | 60/147 (40.8) | -25.9 (-39.2, -12.6) | 0.65 (0.50, 0.85) | 0.001 |
| **Satisfied with life [5 stars]** | 368/581 (63.3) | 368/561 (65.6) | 2.3 (-3.3, 7.9) | 1.03 (0.96, 1.11) | 0.429 |
| **Not thin/severely thin** | 562/584 (96.2) | 544/560 (97.1) | 0.9 (-1.2, 3.0) | 1.01 (1.00, 1.02) | 0.203 |
| **Not obese** | 560/584 (95.9) | 537/560 (95.9) | 0.0 (-2.3, 2.3) | 1.00 (0.99, 1.01) | 0.975 |
| **No anaemia** | 521/577 (90.3) | 513/559 (91.8) | 1.5 (-1.8, 4.8) | 1.02 (0.99, 1.05) | 0.257 |
| **No severe anaemia** | 576/577 (99.8) | 558/559 (99.8) | 0.0 (-0.5, 0.5) | 1.00 (0.99, 1.00) | 0.379 |
| **No school missed due to menstruation in the past month** | 385/507 (75.9) | 397/479 (82.9) | 7.0 (2.0, 12.0) | 1.08 (1.03, 1.14) | 0.004 |
| **No school missed due to illness in the past month** | 281/581 (48.4) | 307/561 (54.7) | 6.3 (0.5, 12.1) | 1.10 (0.99, 1.21) | 0.065 |
| **School results OK/good/excellent** | 533/574 (92.9) | 495/559 (88.6) | -4.3 (-7.7, -0.9) | 0.96 (0.93, 0.99) | 0.009 |
| **Health did not impair school performance in past 4 months** | 351/581 (60.4) | 365/561 (65.1) | 4.7 (-0.9, 10.3) | 1.06 (0.98, 1.14) | 0.134 |
| **Health did not impair school participation in past 4 months** | 396/581 (68.2) | 413/561 (73.6) | 5.4 (0.1, 10.7) | 1.07 (1.01, 1.14) | 0.029 |
| **Health checks are worthwhile** | 350/581 (60.2) | 364/561 (64.9) | 4.7 (-0.9, 10.3) | 1.08 (0.99, 1.17) | 0.077 |

See Supplementary Table 5 for secondary outcomes definitions

*[1] Prevalence ratios derived from population-averaged generalised linear models adjusted for within-location correlation.

(-) Prevalence ratio and respective p-value not calculated for secondary outcomes with sparse data (log-binomial model did not converge)

*Supplementary Table 3d: before-and-after comparison of prevalence of specific conditions/behaviors (female aged 15-19)*

| | Baseline n/N (%) | Follow-up n/N (%) | Difference in proportions (95% CI) | PR (95% CI) | p-value |
|---|---|---|---|---|---|
| **Health knowledge quiz scores >= 4/8** | 176/597 (29.5) | 110/488 (22.5) | -7.0 (-12.2, -1.8) | 0.76 (0.63, 0.92) | 0.005 |
| **Strong desire to avoid drugs/alcohol** | 508/597 (85.1) | 434/488 (88.9) | 3.8 (-0.2, 7.8) | 1.04 (1.00, 1.09) | 0.045 |
| **Strong desire to be active** | 458/597 (76.7) | 404/488 (82.8) | 6.1 (1.3, 10.9) | 1.07 (1.02, 1.13) | 0.005 |
| **Empowered to make health-related decisions** | 222/597 (37.2) | 197/488 (40.4) | 3.2 (-2.6, 9.0) | 1.09 (0.96, 1.24) | 0.207 |
| **Has sugary drink less than once per day** | 487/597 (81.6) | 339/488 (69.5) | -12.1 (-17.2, -7.0) | 0.85 (0.80, 0.91) | 0.000 |
| **Eats fruit at least once per day** | 174/597 (29.1) | 160/488 (32.8) | 3.7 (-1.8, 9.2) | 1.12 (0.95, 1.32) | 0.184 |
| **Brushes teeth with toothpaste at last once per day** | 546/599 (91.2) | 453/488 (92.8) | 1.6 (-1.6, 4.8) | 1.01 (0.98, 1.05) | 0.457 |
| **Sleeps at least 8 hours** | 444/599 (74.1) | 342/488 (70.1) | -4.0 (-9.4, 1.4) | 0.96 (0.90, 1.03) | 0.218 |
| **Physically active every/almost every day in past 2 weeks** | 244/599 (40.7) | 236/488 (48.4) | 7.7 (1.8, 13.6) | 1.19 (1.07, 1.34) | 0.002 |
| **Low or no substance use (CRAFFT score <2)** | 551/599 (92.0) | 469/488 (96.1) | 4.1 (1.3, 6.9) | 1.05 (1.02, 1.07) | 0.001 |
| **No smoking in past 30 days** | 588/599 (98.2) | 482/488 (98.8) | 0.6 (-0.8, 2.0) | - | - |
| **Had support with mental health challenges** | 76/98 (77.6) | 60/76 (78.9) | 1.3 (-11.0, 13.6) | - | - |
| **Had <=1 sexual partner in past 6 months** | 335/345 (97.1) | 258/266 (97.0) | -0.1 (-2.8, 2.6) | 1.00 (0.97, 1.02) | 0.769 |
| **Uses condoms most/all the time** | 22/54 (40.7) | 6/31 (19.4) | -21.3 (-40.4, -2.2) | 0.56 (0.27, 1.16) | 0.119 |
| **Using effective contraception** | 35/58 (60.3) | 19/34 (55.9) | -4.4 (-25.3, 16.5) | 0.92 (0.65, 1.30) | 0.641 |
| **Linked to care if HIV+** | 17/23 (73.9) | 19/25 (76.0) | 2.1 (-22.4, 26.6) | 1.06 (0.96, 1.17) | 0.242 |
| **Attended clinic in past 4 months [if sick and wanted to go]** | 27/60 (45.0) | 18/34 (52.9) | 7.9 (-13.1, 28.9) | 1.24 (0.83, 1.87) | 0.294 |
| **Satisfied with life [5 stars]** | 244/597 (40.9) | 235/488 (48.2) | 7.3 (1.4, 13.2) | 1.16 (1.03, 1.31) | 0.013 |
| **Not thin/severely thin** | 592/599 (98.8) | 476/479 (99.4) | 0.6 (-0.5, 1.7) | - | - |
| **Not obese** | 584/599 (97.5) | 460/479 (96.0) | -1.5 (-3.7, 0.7) | 0.99 (0.97, 1.00) | 0.055 |
| **No anaemia** | 473/595 (79.5) | 426/488 (87.3) | 7.8 (3.4, 12.2) | 1.09 (1.05, 1.14) | 0.000 |
| **No severe anaemia** | 591/595 (99.3) | 486/488 (99.6) | 0.3 (-0.6, 1.2) | 1.01 (1.00, 1.01) | 0.277 |
| **No school/work missed due to menstruation in the past month** | 356/591 (60.2) | 238/412 (57.8) | -2.4 (-8.6, 3.8) | 0.96 (0.88, 1.04) | 0.302 |
| **No school/work missed due to illness in the past month** | 266/495 (53.7) | 204/414 (49.3) | -4.4 (-10.9, 2.1) | 0.91 (0.81, 1.02) | 0.116 |
| **School results OK/good/excellent** | 418/514 (81.3) | 318/383 (83.0) | 1.7 (-3.4, 6.8) | 1.03 (0.98, 1.08) | 0.326 |
| **Health did not impair school/work performance in past 4 months** | 243/597 (40.7) | 243/488 (49.8) | 9.1 (3.2, 15.0) | 1.15 (1.03, 1.27) | 0.010 |
| **Health did not impair school/work participation in past 4 months** | 316/597 (52.9) | 292/488 (59.8) | 6.9 (1.0, 12.8) | 1.09 (1.01, 1.17) | 0.022 |
| **health checks are worthwhile** | 434/597 (72.7) | 360/488 (73.8) | 1.1 (-4.2, 6.4) | 1.01 (0.95, 1.08) | 0.745 |

See Supplementary Table 5 for secondary outcomes definitions

*[1] Prevalence ratios derived from population-averaged generalised linear models adjusted for within-location correlation.

(-) Prevalence ratio and respective p-value not calculated for secondary outcomes with sparse data (log-binomial model did not converge)

*Supplementary Table 4- Conditions and risk behaviors screened for and management indicated if the participant screened positive*

| **Condition, issue or behavior *(grouping)*** | **Population** | **Screening tool** | **Flag** | **Management** | |
|---|---|---|---|---|---|
| | | | | *Everyone who is flagged* | *Only if indicated by nurse and/or counsellor* |
| **Home *(psychosocial)*** | All | Self-completion questionnaire (HEEADSSS) | Doesn't get along with the people they live with, or has no one at home to talk to about problems | Counselling/ advice by nurse and/or counsellor | Referral |
| **School/work *(psychosocial)*** | All | Self-completion questionnaire (HEEADSSS) | School/workplace is never safe, or has no one at school/work they can talk to about problems or reports school results are bad | Counselling/ advice by nurse and/or counsellor | Referral |
| **Body *(psychosocial)*** | All | Self-completion questionnaire (HEEADSSS) | The look of their body makes them feel angry or sad, or they don't think their weight is healthy | Counselling/ advice by nurse and/or counsellor | Referral |
| **Meals *(psychosocial)*** | All | Self-completion questionnaire | Missed dinner (main meal of the day) more than once in past 2 weeks | Counselling/ advice by nurse and/or counsellor | Referral |
| **Friends *(psychosocial)*** | All | Self-completion questionnaire (HEEADSSS) | Relationship with friends is bad, or is bullied often/always | Counselling/ advice by nurse and/or counsellor | Referral |
| **Common mental health disorders** | All | Self-completion questionnaire 10-14 year olds: PSC-Y-17 | 10-14 year olds: Paediatric Symptom Checklist-Y-17 score ≥15 (ref) | Counselling/ advice by nurse and/or counsellor | Referral |

| | | | | | |
|---|---|---|---|---|---|
| | | Self-completion questionnaire 15-19 year olds: PHQ-9 and GAD-7 | 15-19 year olds: Patient Health Questionnaire- 9 (PHQ-9) score ≥10 (ref) or Generalised Anxiety Disorder questionnaire (GAD-7) score ≥10 (ref) (ref) | Counselling/ advice by nurse and/or counsellor | Referral |
| **Suicide risk** | All | Self-completion questionnaire (PHQ-9/PHQ-A) | Had serious thoughts about ending their life at least once in the past month (10-19 years) (Patient Health Questionnaire for Adolescents (PHQ-A)) (ref)<br>or<br>Had thoughts that would be better off dead or of hurting themselves in some way on several days or more in the past two weeks (15-19 years only) (PHQ-9) (ref) | Counselling/ advice by nurse and/or counsellor | Referral |
| **Alcohol and substance use *(substance use/smoking)*** | All | Self-completion questionnaire. CRAFFT 2.1 | CRAFFT 2.1 (Car, Relax, Alone, Forget, Friends, Trouble) score ≥2 or CRAFFT 2.1 score <2 & report have used alcohol or substances at least once in the past year (ref) (ref) | Counselling/ advice by nurse and/or counsellor | Referral |
| **Smoking *(substance use/smoking)*** | All | Self-completion questionnaire (Global School-based Student Health (GSHS) 2021 modified) | Smoked cigarettes or used another tobacco product or used a vaping product containing nicotine in the past 30 days (ref) | Counselling/ advice by nurse and/or counsellor | Referral |
| **Poor oral hygiene** | All | Self-completion questionnaire (GSHS 2021) | Brushed teeth less than daily or without flouride toothpaste (ref) | Nurse counselling | |
| **Poor sleep** | All | Self-completion questionnaire | Less than 8 hours sleep per night (ref) | Counselling/ advice by nurse and/or counsellor | |
| **Limited exercise** | All | Self-completion questionnaire | Was physically active 'once' or 'never' in past 2 weeks (ref) | Counselling/ advice by nurse | |

| | | | | | |
|---|---|---|---|---|---|
| | | (GSHS 2021 modified) | | and/or counsellor | |
| **Epilepsy** | All | Self-completion questionnaire | Ever has fits or episodes when legs or arms have jerking movements or fall to the ground and lose consciousness (ref) | Nurse counselling + referral | |
| **Sexual risk** | 16-19 years in the community only | Self-completion questionnaire (GSHS modified) | Two or more sexual partners in last 6 months, or uses condoms never/rarely/sometimes, or is male and attracted to men (among those who report having had at least one sexual partner in the past 6 months) (ref) | Counselling /advice by nurse and/or counsellor | |
| **Uncircumcised male** | 16-19 years males in the community only | Self-completion questionnaire & visual inspection (if required for confirmation) (Demographic Health Survey modified) | Uncircumcised male (ref) | Referral | |
| **Family Planning need** | 16-19 years in the community only | Self-completion questionnaire (GSHS modified) | Sexually active, self-reported not pregnant (females) or partner not pregnant (males), and not using effective contraception or either reported using or that their partner used emergency contraception at least once in the past 6 months (ref) | Nurse counselling | Contraception provision (COC, POP, Depo-Provera, emergency contraception, condoms) or referral for long-acting reversible contraception |
| **HIV+ and not engaged in care *(HIV)*** | 16-19 years in the community only | Self-reported questionnaire | Reported being HIV positive and not registered with a clinic that provides HIV care or not taking ART | Counselling/advice by nurse and/or counsellor | |
| **Sexually Transmitted** | 16-19 years in the community only | Self-completion questionnaire | Reported symptoms of STIs with nurse review and physical examination to confirm diagnosis. Syndromic STIs: | STI syndromic management | Referral |

<table>
<tr><td>Infections symptoms<br>(STI)</td><td></td><td></td><td>- Urethral discharge and dysuria in men<br>- Acute epididymo-orchitis in men<br>- Balanitis or Balano-posthitis in men<br>- Vaginal discharge in women<br>- Pelvic inflammatory disease<br>- Genital ulcers<br>- Bubo</td><td></td><td></td></tr>
<tr><td>Hearing impairment</td><td>All</td><td>hearScreen® smartphone screening tool (ref) . Ear examination.</td><td>Mild to profound hearing loss based on pure tone average ≥ 26dB (ref)</td><td>Ear examination with ear wax removal and repeat screening if ear wax removed</td><td>Referral</td></tr>
<tr><td>Visual impairment</td><td>All</td><td>Peek Acuity smartphone app (ref)</td><td>Distance vision ≤6/9.5 in either eye (ref)</td><td>Referral</td><td></td></tr>
<tr><td>Physical impairment-lower limb<br>(Physical impairment)</td><td>All</td><td>Standing broad jump test</td><td>Best (longest) of two jump test measurements ≤10th percentile of adolescent norms (<80 cm) (ref)</td><td>Counselling/advice by nurse and/or counsellor</td><td>Referral</td></tr>
<tr><td>Physical impairment-upper limb<br>(Physical impairment)</td><td>All</td><td>Grip strength test</td><td>Best (highest) of grip test measurements in either arm ≤10th percentile of adolescent norms (ref)<br><u>Right hand</u>
<table>
<tr><td></td><td colspan="2">Right arm</td><td colspan="2">Left arm</td></tr>
<tr><td>Age</td><td>Males less than (kgs)</td><td>Females less than (kgs)</td><td>Males less than (kgs)</td><td>Females l than (kgs</td></tr>
<tr><td>10</td><td>10.8</td><td>10.4</td><td>9.1</td><td>9.4</td></tr>
<tr><td>11</td><td>12.9</td><td>12.5</td><td>11.2</td><td>11.2</td></tr>
<tr><td>12</td><td>15.8</td><td>14.6</td><td>14.1</td><td>13.0</td></tr>
<tr><td>13</td><td>19.1</td><td>16.5</td><td>17.9</td><td>14.6</td></tr>
<tr><td>14</td><td>22.7</td><td>18.0</td><td>22.1</td><td>15.8</td></tr>
<tr><td>15</td><td>26.0</td><td>18.9</td><td>26.1</td><td>16.7</td></tr>
<tr><td>16</td><td>28.7</td><td>19.3</td><td>29.2</td><td>17.3</td></tr>
<tr><td>17</td><td>30.7</td><td>19.3</td><td>31.2</td><td>17.8</td></tr>
<tr><td>18</td><td>34.1</td><td>19.3</td><td>34.1</td><td>18.2</td></tr>
<tr><td>19</td><td>35.7</td><td>19.3</td><td>35.7</td><td>19.2</td></tr>
</table></td><td>Counselling/advice by nurse and/or counsellor</td><td>Referral</td></tr>
</table>

<table>
<tr><th></th><th></th><th></th><th></th><th></th><th></th></tr>
<tr><td>Oral health condition</td><td>All</td><td>Oral examination of teeth and gums</td><td>Presence of caries, cavities, or gum disease on oral examination (ref)</td><td>Referral</td><td></td></tr>
<tr><td>Anaemia</td><td>All</td><td>Finger prick haemoglobin (Hb) measurement using Hemocue Hb301 machine (ref)</td><td>Haemoglobin indicative of mild, moderate and severe anaemia (WHO guideline) (ref)<br><br>
<table>
<tr><th><u>Age</u></th><th><u>Normal</u></th><th><u>Mild-Moderate anaemia</u></th><th><u>Severe anaemia</u></th></tr>
<tr><td>≤11 yrs</td><td>Female: Hb ≥11.5g/dL</td><td>Female: Hb <11.5g/dL</td><td>Female: Hb <8.0g/dL</td></tr>
<tr><td></td><td>Male: Hb ≥11.5g/dL</td><td>Male: Hb <11.5g/dL</td><td>Male: Hb <8.0g/dL</td></tr>
<tr><td>12-14 yrs</td><td>Female: Hb ≥12.0g/dL</td><td>Female: Hb <12.0g/dL</td><td>Female: Hb <8.0g/dL</td></tr>
<tr><td></td><td>Male: Hb ≥12.0g/dL</td><td>Male: Hb <12.0g/dL</td><td>Male: Hb <8.0g/dL</td></tr>
<tr><td>≥15 yrs</td><td>Female: Hb ≥12.0g/dL</td><td>Female: Hb <12.0g/dL</td><td>Female: Hb <8.0g/dL</td></tr>
<tr><td></td><td>Male: Hb ≥13.0g/dL</td><td>Male: Hb <13.0g/dL</td><td>Male: Hb <8.0g/dL</td></tr>
<tr><td>Pregnant (≥15 yrs)</td><td>Hb ≥11.0g/dL</td><td>Hb <11.0g/dL</td><td>Hb <8.0g/dL</td></tr>
</table></td><td>3 months iron-folic acid supplementation for mild, moderate or severe anaemia and, if severe anaemia, referral</td><td></td></tr>
<tr><td>Underweight<br>Overweight<br>Obese<br>(Malnutrition)</td><td>All</td><td>Height and weight measurement</td><td>Body Mass Index (BMI) for sex and age indicates severe thinness (-3 Standard Deviations (SD)), thinness (-2SD), overweight (+1SD) or obese (+2SD) https://www.who.int/tools/growth-reference-data-for-5to19-years/indicators/bmi-for-age</td><td>Nurse counselling for all four conditions + referral for severe thinness, thinness or obese only</td><td></td></tr>
<tr><td>Schistosomiasis</td><td>All</td><td>Schistosoma haematobium urine filtration test -Urine sample</td><td>Tests positive for S Haematobium (ref)</td><td>Treatment with praziquantel</td><td></td></tr>
<tr><td>Elevated blood pressure</td><td>15-19 year olds only</td><td>Blood pressure measured using digital sphygmomanometer. Two measurements.</td><td>Second BP measurement (Elevated blood pressure, Stage 1 hypertension, Stage 2 hypertension, or Hypertensive crisis) (ref)<br><br>Normal BP: < 120/< 80 mmHg<br>Elevated BP: 120/< 80 to 129/< 80 mmHg<br>Stage 1 HTN: 130/80 to 139/89 mmHg</td><td>3rd BP measurement by nurse<br>Nurse counselling</td><td></td></tr>
</table>

| | | | | | |
|---|---|---|---|---|---|
| | | | Stage 2 HTN: 140/90 to 179/119 mmHg<br>Hypertensive Crisis: >=180/120 mmHg | If hypertensive crisis or pre-eclampsia then referral | |
| **STI- CT/NG**<br>***(STI)*** | 16-19 years in the community only | Urine sample tested for *Chlamydia trachomatis (CT)* and *Neisseria gonorrheae (NG)* using GeneXpert Xpert CT/NG. | Tested positive for CT or NG (ref) | Treatment on a subsequent day when laboratory results were available as per national guidelines | |
| **STI- TV**<br>***(STI)*** | 16-19 year old females in the community only | Self-taken vaginal swab tested for *Trichomonas vaginalis (TV)* with OSOM® Trichomonas Test | Tested positive for TV (females only) (ref) | Treatment on-the-spot | |
| **HIV**<br>***(HIV)*** | 16-19 years in the community only | HIV oral mucosal self-test (OraQuick HIV-1/2 Test Kit) +/- confirmatory blood-based rapid diagnostic test (Alere Determine HIV-1/2 Test Kit/Chembio HIV-1/2 Test Kit) | Tests positive with OraQuick and confirmatory test using Alere Determine rapid diagnostic test (ref) (ref) | Counselling/advice by nurse and/or counsellor and referral | |
| **Mid-upper arm circumference (MUAC)** | All | | Not flagged | N/A | |
| **Waist circumference** | All | | Not flagged | N/A | |
| **Finger tapping** | All | | Not flagged | N/A | |

HEEADSSS: Home environment, Education/employment, Eating, peer-related Activities, Drugs, Sexuality, Suicide/depression, and Safety[1]

*Supplementary Table 5 Evaluation outcomes*

| Outcome | Method of assessment | Source(s) of data |
|---|---|---|
| **Primary outcome** | | |
| Proportion of those screening positive for at least one condition who received appropriate on-the-spot care or complete appropriate referral for all identified conditions by the time of their follow-up visit | Denominator<br>Screening positive was defined as receiving a warning flag for a condition during check-up visit (Table S4), and the warning flag was not redefined as a 'false warning flag' by the study nurse<br><br>Numerator<br>During the screening visit the 'action taken' by the nurse was recorded. Potential actions included treatment, counselling, information provision and/or referral. Each condition has a pre-defined 'appropriate on-the-spot care' action or potential actions (Table S4). Where multiple actions are required then all actions should have been taken e.g. for severe anemia the client should have been treated AND referred. Participants had completed the appropriate referral if they had attended at least one referral visit by the time of the follow-up visit*<br><br>If the participant was offered but refused treatment or refused to discuss the issue then they were considered not to have received appropriate on-the-spot care<br><br>Participants already receiving care for their condition and not requiring further intervention were considered to have received appropriate on-the-spot care<br><br>*When finalising the statistical analysis plan prior to the follow-up data collection, the eligible time period for attendance at the first referral appointment was amended from 'within 4-months' (Banati et al, 2024) to 'by the time of the follow-up visit' as there had been some operational delays in the timing of the follow-up visits | Y-Check screening application data<br>Laboratory test results<br>Paper referral forms<br><br>Correct on-the-spot care for each condition was defined in the manual of operations and actions were recorded by the nurse in the screening app<br><br>Completion of appropriate referral was measured by collecting completed referral forms from the referral service providers |
| **Secondary implementation outcomes** | | |
| Proportion of those screening positive for each condition who received appropriate on-the-spot care or completed appropriate referral for that condition by the time of their follow-up visit | As for primary outcome above | As for primary outcome above |
| Yield of previously untreated conditions | Numerator: number of individuals identified with a condition or risk behavior that was not currently being treated and required counselling, further investigation and/or treatment<br><br>Denominator: individuals who were screened for that condition/behavior | Y-Check screening app data<br><br>Laboratory or physical test results |

| Client outcomes | | |
|---|---|---|
| Knowledge about health services and health behaviors | Health knowledge was measured using an 8-item quiz:<br>1. Which of the following are effects of substance use? (Multiple answers, 3 true, 1 false)<br>2. Which of the following are effects of drinking alcohol? (Multiple answers, 3 true, 1 false)<br>3. How often should a person brush their teeth with fluoride toothpaste daily? (Single answer)<br>4. Which of the following are good ways to help yourself to stay well when you are feeling sad or anxious? (Multiple answers, 2 true, 3 false)<br>5. How many hours of sleep should you be getting to function properly throughout the day? (Single answer)<br>6. How long per day should you spend doing physical activity, such as walking fast, running, playing sports? (Single answer)<br>7. How often should you eat fruit? (Single answer)<br>8. Which of these is a good example of a healthy snack? (Single answer)<br><br>10-14 year-old participants were not invited to answer questions 1 and 2 so their knowledge score was calculated based on questions 3 to 8. Participants scored 1 point for each correct answer, for a maximum of 6. Multiple-choice answers had to be entirely correct to score a point.<br>For 10-14 year-olds, the proportion of participants who scored ≥3/6 is reported.<br>For 15-19 year-olds, the proportion of participants who score ≥4/8 is reported. | Self-completed evaluation questionnaire at follow-up vs. at the check-up (baseline) |
| Intentions to adopt healthy behavior | Each one has the responses 'strong desire, 'some desire, 'no desire'.<br>• Q901. Desire/intention to avoid drugs (15-19y only)<br>• Q902. Desire/intention to avoid drinking alcohol (15-19y only)<br>• Q903. Desire/intention to be more active<br><br>For 10-14y the proportion of clients who report strong desire to be more active will be reported. For 10-14y this question was asked at 4 months only | Self-completed evaluation questionnaire only at the follow-up visit (10-14y) or at the follow-up visit and the check-up visit (baseline) (15-19y) |
| Agency to make decisions about health and wellbeing | Agency to make decisions about health was measured among 15-19 year old participants using 2 items:<br>• Q801. Do you feel empowered to make your own health-related choices/decisions? (Absolutely not, sometimes, definitely) The proportion of clients who responded 'definitely' was reported.<br>• Q802, 'How easy would you say it is to make decisions to improve your health?' (Very difficult, fairly difficult, fairly easy). The proportion who responded 'fairly easy' was reported | Self-completed evaluation questionnaire |
| Health-related risk and protective behaviors | | Self-reported screening tool; Self-completed evaluation questionnaire |
| Frequency of sugary drink consumption | The proportion who reported consuming a sweetened drink at least once a day in the past week | Self-reported screening tool and follow-up questionnaire |

| | | |
|---|---|---|
| Frequency of fruit consumption | The proportion who reported eating fruit at least once a day in the past week | Self-reported screening tool and follow-up questionnaire |
| Oral hygiene | The proportion who reported cleaning their teeth with flouride toothpaste at least once a day in the past 30 days | Self-reported screening tool and follow-up questionnaire |
| Sleep duration | The proportion who reported that they slept on average for-at least 8 hours on weekdays. Participants recorded the average time they went to bed and the average time they got up | Self-reported screening tool and follow-up questionnaire |
| Physical activity | The participant reported how many days they were physically active for more than 1 hour in the past 2 weeks (never, once, almost every day, or every day). The proportion who were active 'almost every day' or 'every day' was calculated | Self-reported screening tool and follow-up questionnaire |
| Alcohol and substance use | Measured using the 6-item CRAFFT screening tool (see Table S4), scored from 0-6 with 6 being highest risk | Self-reported screening tool and follow-up questionnaire |
| Smoking | The proportion of participants who have not smoked any cigarettes, other tobacco products or vaping devices in the previous 30 days | Self-reported screening tool and follow-up questionnaire |
| Has support with mental health challenges | The proportion of participants experiencing mental health challenges in the past 4 months who took any action to support their mental health (talked to someone, got enough sleep, exercised, used a hotline, online help or counselling) | Self-reported screening tool and follow-up questionnaire |
| Number of sexual partners | The proportion who reported none or one sexual partner in the past 6 months | Self-reported screening tool and follow-up questionnaire |
| Condom use | Among those who reported that they have had sex in the past 6 months, the proportion who reported using condoms always/most of the time | Self-reported screening tool and follow-up questionnaire |
| Effective contraception use | Proportion of those sexually active in the past 6 months who were not pregnant (partner not pregnant) and who were using effective contraception (male condom, female condom, oral contraceptive pill, implant, injection, or IUD loop) | Self-reported screening tool and follow-up questionnaire |
| Linked to care if HIV+ | Proportion of adolescents who were HIV positive and were linked to care and currently on ART. Knowledge of HIV status was defined as participants whose reported HIV status (positive or negative) agreed with the result of their HIV test at the check-up visit. Clients who self-reported as HIV positive but whose test result was negative were coded as HIV negative and as not knowing their HIV status | Self-reported screening tool and follow-up questionnaire |
| Engagement with health and other services within the past 4 months | The proportion who attended a clinic in the past 4 months if they were sick and reported that they wanted to go to the clinic | Self-completed baseline and follow-up questionnaires |

| | | |
|---|---|---|
| Life satisfaction | The proportion who responded 5/5 stars to the following question: 'Overall, how satisfied are you with life as a whole these days?" | Self-completed baseline and follow-up questionnaires |
| Self-esteem | Self-esteem was measured with the 10-item Rosenberg scale in secondary schools and community venues. The scale ranges from 10 (poor) to 40 (excellent) with 40 being the highest | Self-completed baseline and follow-up questionnaires |
| Quality of life | Quality of life was measured using the Child Health Utility instrument (CHU9D), a 9-item paediatric generic preference-based measure of health-related quality of life. Scores range from 0-40. Australian adolescent weights were used | Self-completed baseline and follow-up questionnaires |
| Improvements in diagnosed conditions | Conditions: STI symptoms, STI test positive, common mental health symptoms, suicide risk, epilepsy, visual impairment, hearing impairment<br><br>Numerator: all those who screened negative for the condition at follow-up<br><br>Denominator: all those who screened positive for the condition at check-up and who participated in the follow-up survey | Y-Check screening application data<br><br>Laboratory or physical test results<br><br>Follow-up clinical data |
| Clinical outcomes | Underweight (see Table S4) | Clinical data (see Table S4) |
| | Obese (see Table S4) | |
| | Anemia (see Table S4) | |
| | Severe anemia (see Table S4) | |
| Educational outcomes | Proportion who missed > =1 days school/work in the previous month due to menstruation, among girls who reported that they had started menstruation | Self-completed baseline and follow-up questionnaires |
| | Proportion who missed > =1 days school/work in the previous month due to ill health | Self-completed baseline and follow-up questionnaires |
| | The proportion who reported that their school results were bad. Response options were: bad/ok/good/excellent | Self-completed baseline and follow-up questionnaires |
| | Proportion who reported that a health condition prevented them doing their best in class | Self-completed baseline and follow-up questionnaires |

| | | |
|---|---|---|
| | Proportion who reported that a health condition prevented their participation in school activities | Self-completed baseline and follow-up questionnaires |
| Perception of relative lack of importance of getting health checked | One client-centred outcome was measured using the following item, rated on a scale of 1-5 (lowest to highest): How worthwhile do you think getting your health checked is?<br>The proportion of participants who gave a score less than 5 has been reported | Self-completed baseline and follow-up questionnaires |

*Supplementary Table 6 Process evaluation data collection methods*

| Data collection Method | Participants | How many/much |
|---|---|---|
| **1. Non-participant observation of Y-Check (including informal chats)** | All Y-Check intervention team and participants in 4 primary and 2 secondary schools and 2 community venues | 24 days of observation delivery across Y-Check intervention settings |
| **2. Non-participant observation of referrals** | Participants referred for referrals and their service providers | 6 observations of vision, hearing, oral health, epilepsy, growth and nutrition and mental health referrals (one each) |
| **3. Non-participant observation of Y-Check debriefing meetings** | Y-Check intervention team | 8 debriefing meetings* |
| **4. In-depth interviews (IDIs)** | Participants who received Y-Check intervention (9); participants who attended referrals (10); referral service providers (5); intervention team members (6); school authorities(6) | 36 IDIs |
| **5. Participatory workshops** | Participatory workshops with adolescents who participated in Y-Check (4), adolescents who didn't participate in Y-Check (4), teachers (2), parents of adolescents who participated in Y-Check (1); parents of adolescents who didn't participate in Y-Check (1). | 12 participatory workshops |
| **6. Self-administered questionnaire** | Participants at completion of Y-Check intervention | 1747 exit interviews |

*Debriefing meetings were 1-2 hour team meetings during which the Y-Check field team updated the study coordinator on progress with the activities and any challenges were discussed.
